# The Effects of Vaccination and Immunity on Bacterial Infection Dynamics *In Vivo*

**DOI:** 10.1371/journal.ppat.1004359

**Published:** 2014-09-18

**Authors:** Chris Coward, Olivier Restif, Richard Dybowski, Andrew J. Grant, Duncan J. Maskell, Pietro Mastroeni

**Affiliations:** University of Cambridge, Department of Veterinary Medicine, Cambridge, United Kingdom; Stanford University School of Medicine, United States of America

## Abstract

*Salmonella enterica* infections are a significant global health issue, and development of vaccines against these bacteria requires an improved understanding of how vaccination affects the growth and spread of the bacteria within the host. We have combined *in vivo* tracking of molecularly tagged bacterial subpopulations with mathematical modelling to gain a novel insight into how different classes of vaccines and branches of the immune response protect against secondary *Salmonella enterica* infections of the mouse. We have found that a live *Salmonella* vaccine significantly reduced bacteraemia during a secondary challenge and restrained inter-organ spread of the bacteria in the systemic organs. Further, fitting mechanistic models to the data indicated that live vaccine immunisation enhanced both the bacterial killing in the very early stages of the infection and bacteriostatic control over the first day post-challenge. T-cell immunity induced by this vaccine is not necessary for the enhanced bacteriostasis but is required for subsequent bactericidal clearance of *Salmonella* in the blood and tissues. Conversely, a non-living vaccine while able to enhance initial blood clearance and killing of virulent secondary challenge bacteria, was unable to alter the subsequent bacterial growth rate in the systemic organs, did not prevent the resurgence of extensive bacteraemia and failed to control the spread of the bacteria in the body.

## Author Summary

The bacterium *Salmonella enterica* causes gastroenteritis and the severe systemic diseases typhoid, paratyphoid fever and non-typhoidal septicaemia (NTS). Treatment of systemic disease with antibiotics is becoming increasingly difficult due to the acquisition of resistance. Licensed vaccines are available for the prevention of typhoid, but not paratyphoid fever or NTS. Vaccines can be either living (attenuated strains) or non-living (e.g. inactivated whole cells or surface polysaccharides) and these different classes potentially activate different components of the host immune system. Improvements in vaccine design require a better understanding of how different vaccine types differ in their ability to control a subsequent infection. We have improved a previously developed experimental system and mathematical model to investigate how these different vaccine types act. We show that the inactivated vaccine can only control bacterial numbers by a transient increase in bactericidal activity whereas the living vaccine is superior as it can induce an immune response that rapidly kills, then restrains the growth and spread of infecting bacteria.

## Introduction

*Salmonella enterica* causes systemic diseases (typhoid and paratyphoid fever) [1], food-borne gastroenteritis and non-typhoidal septicaemia (NTS) [2]–[4] in humans and in many other animal species world-wide. Current measures to control *S. enterica* infections are sub-optimal. The emergence of multi-drug resistant strains has reduced the usefulness of many antibiotics [5]–[6]. Prevention of infection of food-production animals by implementation of biosecurity or hygiene measures is expensive and is undermined by increased free-range production.

Vaccination remains the most feasible means to counteract *S. enterica* infections. There is an urgent need for improved vaccines against typhoid fever and there are currently no licensed paratyphoid or NTS vaccines [7]. To attain a high level of protective immunity against systemic infections with virulent strains of *Salmonella* in susceptible hosts it is necessary to induce both antibody responses and T-helper type 1 (T_H_1) cell-mediated immunity [8]. This is due to the fact that intracellular control requires T_H_1 immunity whereas antibodies can only target the bacteria in the extracellular compartment (reviewed in [9]–[10]).

New generations of live attenuated vaccines have been constructed in the last two decades and are currently being evaluated in field trials. These vaccines mimic the course of natural infection and are more protective than previous ones, but we do not understand the mechanisms responsible for this [11]–[12]. There is also a recent trend towards the development of non-living vaccines against *S. enterica* enteric diseases for humans and other animals. Current non-living vaccines are based on inactivated whole cells and surface polysaccharides (e.g. Vi polysaccharide and Vi conjugate vaccines for humans) [13]–[14] and subunit protein-based vaccines are being considered. However, non-living *S. enterica* vaccines vary greatly in their protective ability [15]–[19]. Vaccine design and selection is still largely an empirical process. This is due to our insufficient understanding of how vaccine-induced immune responses impact precisely on the dynamics of a secondary infection in terms of bacterial division, killing, spread and persistence in the tissues.

Interactions between infectious agents and their hosts occur in diverse environments and over a range of scales: from initial contact at the single cell level; spread throughout various compartments of the host; and between hosts at a population level. Intervention strategies to control infections can interfere with the host-pathogen relationship at all these levels so understanding the dynamics of infections at all scales is important. Mathematical approaches have been extensively used to model infection dynamics on the population level but until relatively recently within-host dynamics have been measured rather crudely, typically by monitoring total pathogen loads in a host or its organs. These measures cannot disentangle the relative contributions of pathogen replication and death to overall growth. For example an unchanging total pathogen load could be due to both replication and killing occurring in balance, or to a lack of replication and no killing. Attempts have been made to measure bacterial pathogen division rates within hosts and cells by techniques such as using non-replicating elements introduced into the bacteria, or dilution of a fluorescent marker that is not expressed within the cell *in vivo* [20]–[23] but these are often confounded by uncertainties concerning the dynamics of these elements in complex *in vivo* milieu and the potential effects on the phenotype of the pathogen being investigated.

We have established and used a research approach based on the tracking of bacterial subpopulations at multiple body sites to study infections of mice and to capture the parameters that govern the intra-organ and inter-organ infection processes. The system employs simultaneous infections with individually identifiable wild-type isogenic tagged strains (WITS) the relative proportions of which can be determined by quantitative real-time PCR (qPCR) [24]–[25] or other molecular techniques. One of the systems to which this technology has been applied is the systemic phase of experimental *Salmonella* infections in mice, a well-established model for invasive disease [25]. In this system, observation of both total bacterial loads and the changes in WITS population structure enabled inference to be drawn on the rates of bacterial replication, killing and spread between organs. This approach allowed us to capture the key dynamic traits of the primary infection process in unimmunised animals and to explore the impact of innate immunity on the infection. In this previous study, we determined that the infection process is multiphasic, with an initial phase of rapid bacterial replication and killing of part of the inoculum by the innate host response mediated by reactive oxygen species (ROS), followed by a phase of growth and intra-organ spread with the stochastic selection of individual bacterial subpopulations. Later the infection moves to a phase in which bacteraemia and the mixing of different subpopulations of bacteria in the organs occurs [25].

In the present study we refined our framework and used it to understand to what extent and at what stages of the infection different vaccine types and different branches of the vaccine-induced host immune response restrain intracellular division and enhance bacterial killing, whether there are changes in the patterns of local or systemic spread in vaccinated/immune animals, whether immunity acts simultaneously or equally on the global bacterial population, and whether there are intra- or inter-organ differences and heterogeneous traits in the control of individual bacterial subpopulations.

## Results

### Vaccination with live but not killed *Salmonella* restrains both bacterial growth and inter-organ spread

Vaccination with either live or killed *Salmonella* is known to increase the rate of blood clearance of an intravenous challenge, result in a bias of segregation of bacteria towards the liver rather than the spleen, and to enhance the reduction in bacterial loads in the spleen and liver that occurs within the first hours [26]–[30]. To investigate the contributions made by bacterial killing and growth to these changes in total bacterial numbers we performed two separate experiments vaccinating mice with either live attenuated *Salmonella* Typhimurium (STm) SL3261 (Live Vaccine group: LV) or acetone-killed STm SL1344 (Killed Vaccine group: KV). Naive control and vaccinated animals (*n* = 9 or 10, see **Table S1**) were then challenged with ∼300 colony forming units (CFU) of STm WITS and net bacterial numbers and WITS proportions were monitored over a period of 72 h post-challenge (timepoints 30 min, 24, 48, 72 h). The challenge dose was chosen as the minimum to yield reliable colonisation of both the liver and spleen of vaccinated animals as determined in pilot experiments described in the Methods section.

**Figure 1:**
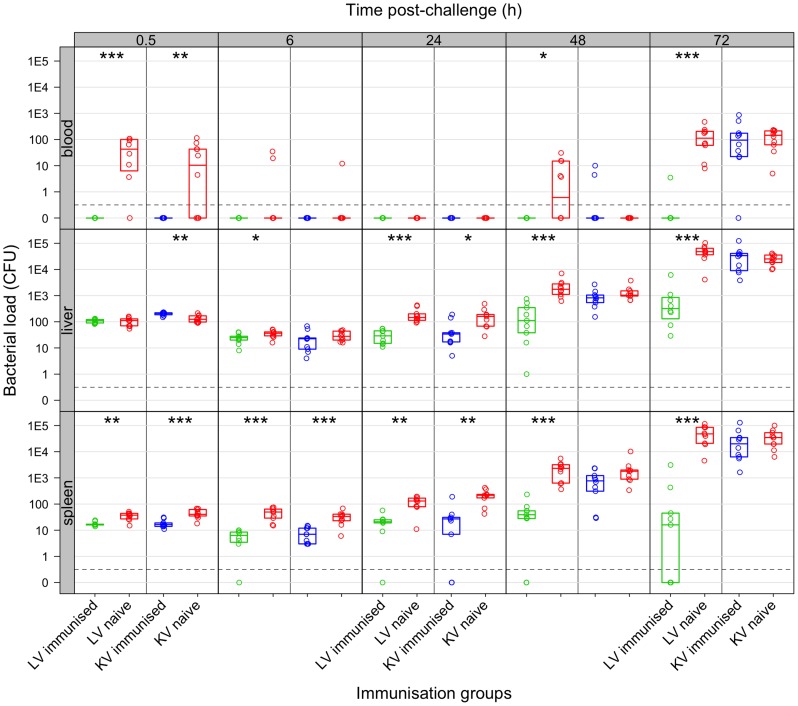
Net growth of secondary challenge bacteria in mice immunised with living or killed vaccine. Live vaccine but not killed vaccine controls bacterial loads following secondary challenge. Bacterial numbers (CFU) in the organs at the indicated times (h) post-challenge in mice immunised with live vaccine (LV: green), killed vaccine (KV: blue) or naive controls (red). Dots show values in each mouse, boxes show the quartiles for each group (25%, median and 75%). Challenge inoculum size doses were: LV experiment 243 CFU of WITS; KV experiment 280 CFU of WITS. Significant differences in bacterial loads compared to naive controls are shown by: *P<0.05; **P<0.01; ***P<0.001 (Mann-Whitney *U*-test).

Vaccination with either type of vaccine resulted in accelerated clearance of the WITS challenge inoculum from the blood with no circulating bacteria detected at 30 min post-challenge (**Figure 1**). In contrast the majority of naive animals still harboured bacteria in the blood at this time. By 6 hr post-infection (p.i.) the majority of vaccinated and non-vaccinated animals had cleared the challenge dose from the blood and at 24 hr no bacteraemia was observed in any of the mice tested.

At 24 h post-challenge, animals vaccinated with either preparation had a lower bacterial load in the organs compared to naive controls but the kinetics of this reduction differed somewhat between vaccine types (**Figure 1**): at 6 h p.i. bacterial loads in both organs of mice immunised with the LV were lower than in the controls whereas for the KV loads were only significantly lower in the spleen at this timepoint; by 24 h p.i. bacterial loads in both vaccinated groups were lower than in the controls. Subsequently between 24 and 72 h after challenge, mice immunised with the KV and the unimmunised control mice both allowed a rapid increase in bacterial numbers (∼10-fold per day), with no statistically significant difference between these groups, and a resurgence of bacteraemia from 48 h p.i. In clear contrast, net bacterial growth was much slower in the tissues of mice vaccinated with live bacteria and bacteraemia was observed in only one of nine mice tested and even then at a very low level; *Salmonella* were absent from the spleens in 4/9 animals by 72 h p.i.

**Figure 2:**
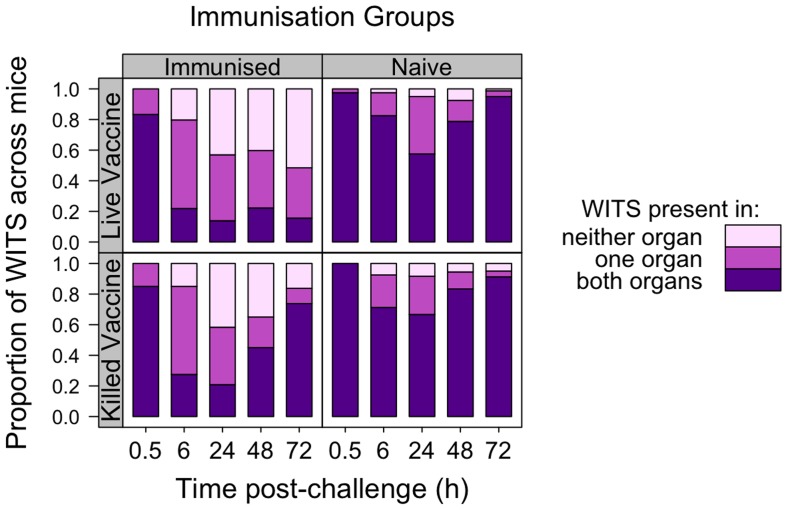
Co-occurrence of WITS in the livers and spleens during secondary challenge. LV-immunised animals show enhanced bactericidal activity, bacteriostasis and reduced inter-organ transfer of bacteria as compared to naive controls. KV-immunised animals show only transient enhancement of bactericidal activity. Each bar shows proportion of WITS present in both liver and spleen (purple), one organ only (dark pink) or neither organ (light pink), pooled across mice within each experimental group at the indicated times (h) post-challenge. Top row: LV; Bottom row: KV; Left column: Immunised animals; Right column: naive animals.

Next we analysed the population structure of WITS in the samples to determine when bacterial death and inter-organ spread occurred. The challenge inoculum contained equal numbers of each of 8 WITS. We first considered the number of distinct WITS per mouse that were present in both organs (liver plus spleen), in one organ only, or in neither of the organs (**Figure 2**). In the absence of bacterial killing we would expect all individual WITS to be represented in one or both organs. In contrast, if bacterial killing was significant then the number of strains present in only one organ, or absent from both would increase. Based on the disappearance of WITS from either organ, we predicted that bacterial death was higher during the first 24 h in animals vaccinated with either vaccine compared to naive mice. In the naive and KV groups, between 48 and 72 h p.i., we observed increases in the numbers of individual WITS that were simultaneously present in both spleen and liver (**Figure 2**). This was suggestive of bacterial transfer between organs and coincided temporally with detection of bacteraemia in these mice from the 48 hr time-point (**Figure 1**). The number of WITS that were present or absent in the organs of mice immunised with the LV remained approximately constant between 24 h and 72 h post-challenge indicating that neither transfer between organs nor substantial bacterial death had occurred in this group of animals.

**Figure 3:**
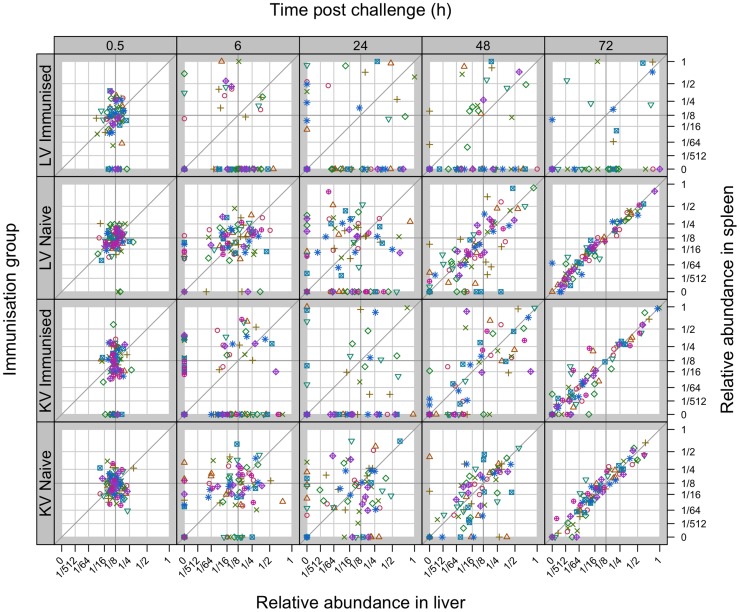
Relative abundance of individual WITS in the spleens and livers. Secondary challenge bacteria segregate into the liver and spleen to form independent populations within 6 h. Subsequently hepatic and splenic populations become homogeneous in naive and KV-immunised animals but not in LV-immunised mice. All WITS within a mouse are represented by the same symbol. Points along the diagonal correspond to equal abundances in both organs. Top two rows: mice immunised with live attenuated *Salmonella* vs naive controls (average challenge dose 243 CFU of WITS); Bottom two rows: mice immunised with acetone-killed *Salmonella* vs naive controls (average challenge dose 280 CFU of WITS).

In addition to determining the presence and absence of WITS from the organs, we measured the proportion of each WITS present in the spleen or liver (**Figure 3**). For the unvaccinated animals at 30 min each WITS could be found in both the spleen and liver with a proportion of approximately 1/8, *i.e.* the population structure was similar to that in the inoculum (which contained equal numbers of the eight WITS). By 6 h p.i. and until the 24 h time-point, the population structures (*i.e.* presence, absence and proportions of each WITS) in each organ of naive mice had diverged (consistent with stochastic killing of bacteria within the organs). By 48 h, coincident with the onset of bacteraemia, the hepatic and splenic WITS population structures became more similar – presumably due to transfer of bacteria between the organs *via* the blood. Between 48 and 72 h p.i. the liver and spleen populations became nearly homogenous within each individual unvaccinated mouse (*i.e.* a given WITS was present in the same proportion in the spleen and liver) (**Figure 3** **;** **Table 1**).

**Table 1:** Increase in correlation between organ bacterial populations over time.

	% positive correlation			
	LV		KV	
Time p.i. (h)	Naive	Immunised	Naive	Immunised
0.5	0	11	0	0
6	0	0	10	10
24	0	0	11	11
48	50	11	56	60
72	100	25	100	100

Percentage of naive, LV or KV-immunised mice exhibiting a statistically significant positive correlation between the abundances of the WITS in the liver and spleen. This was assessed by computing 95% confidence intervals for the coefficients of correlation (see **Figure S7**). Mice with bacteria absent from both liver and spleen were excluded from the analysis.

For animals vaccinated with either LV or KV, there was a bias in segregation of bacteria towards the liver, shown by compression of the points around the vertical in **Figure 3**, and enhanced bactericidal activity resulted in a number of WITS being absent from one or both organs at 6 hr post-challenge. From 48 h the WITS population structures in the KV and LV groups diverged: the KV immunised animals showed a similar pattern to the unvaccinated controls with highly correlated WITS organ populations in all animals by 72 h (**Figure 3** **;** **Table 1**), and with an increase in the number of WITS simultaneously present in both the liver and spleen (**Figure 2**); in contrast, for the LV group there was no increase in the co-occurrence of WITS in both organs (**Figure 2**) and highly correlated WITS organ populations were only observed in a minority of animals (**Figure 3** **;** **Table 1**), therefore indicating that in most of these animals significant inter-organ spread had not occurred up to 72 h post-challenge. The bias in bacterial numbers towards the liver that was observed earlier in the infection was still present in the LV group at this late timepoint, presumably as a consequence of the restraint of both bacterial growth and spread in this group resulting in populations similar to the early timepoints. In contrast for the KV group this bias had disappeared, likely a consequence of uncontrolled spread and growth of bacteria in these animals.

### Mathematical modelling and estimation of the rates of bacterial killing and division in the spleen and liver

The enhanced reduction in total bacterial numbers, the fluctuations in the WITS population structure and the disappearance of WITS from the spleen and liver indicated that the overall dynamics of bacterial division and death are different in naive mice and in vaccinated animals in the early stages of the infection. Bacterial dynamics were described using a stochastic model that keeps track of the number of copies of a single WITS in the blood, liver and spleen simultaneously [9], [25]. The parameters of the model (inoculum size, rates of bacterial replication, killing and transfer from the blood to the organs) were all estimated by fitting the model to the data from each experimental group at 0.5, 6 and 24 h post-inoculation, using maximum likelihood. In order to allow for the possibility of early killing or inactivation of bacteria before they start to colonise the organs, we estimated an effective inoculum size consistent with the total number of bacteria 30 min p.i. in each experimental group. By comparing these values with the average inoculum doses actually used we obtained an estimate of the fraction of bacteria eliminated in the very early stage of infection (*i.e.* within 30 min of inoculation): this fraction was highest (44%) in the LV group (**Table 2**). Biologically this could be a consequence of bacterial killing and/or entry into a non-replicative state [21], [31]–[32]. In our model the remaining bacteria settle into the liver and spleen where they undergo a process that involves replication and killing. Comparing the model estimates for these processes between groups (**Table 2**) we see that for both vaccine types, the model captures the enhanced blood clearance and increase in the proportion of bacteria going to the liver that was observed in the data.

**Table 2:** Maximum-likelihood estimates of model parameters for early events post-challenge.

Experiment	Group	% inoculum removed	Clearance (h)	%liver
A	Naive	6	2.45	75
	LV	44	0.32	86
B	Naive	24	1.3	76
	KV	10	0.34	93

Percentage of the inoculum removed; time taken for 99% of the remaining bacteria to be cleared from the blood into the organs; percentage of the inoculum that segregated to the liver. LV: living vaccine; KV: killed vaccine.

**Figure 4:**
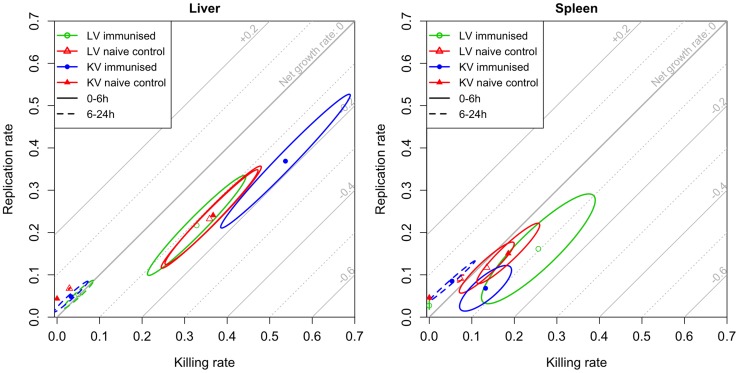
Model estimates for rates of bacterial replication and killing. Rapid bacterial replication and killing is observed over the first 6 h following re-challenge; between 6 and 24 h these rates decrease showing that bacteriostatic mechanisms predominate. Maximum likelihood estimates with 95% confidence ellipses for the killing and replication rates of bacteria in the liver (left) and spleen (right) in each experimental group for the periods 0–6 h (solid ellipses) and 6–24 h (dashed ellipses). The rates are expressed as 1og_10_(CFU) gained or lost per hour (for example, a killing rate of 0.2 means that 37% of bacteria are killed within an hour). Diagonal grid lines indicate net growth rates (replication rate-killing rate).

Estimates of the intra-organ replication and killing rates (**Figure 4**) were very similar in naive mice and in mice immunised with the LV in the first 6 h, whereas higher rates of bacterial replication and killing in the liver were estimated in mice immunized with the KV during this initial period of the secondary infection, resulting in a more rapid net reduction in bacterial numbers. All groups exhibited a large reduction in both the killing and replication rates in the liver after 6 h, with replication rates marginally higher than killing rates. The estimated killing and replication rates were lower in the spleen than in the liver for the first 6 h of the secondary infection, but became similar in both organs between 6 and 24 h. In all cases, the fitted models predicted distributions of bacterial loads and WITS abundancies similar to the data (see Supporting Information **Text S1**)

Thus, based on variations in the population structure of WITS, our model predicts that the reduction in the total numbers of viable bacteria that is seen in the first 24 h in both groups of immunised mice is largely due to enhanced bacterial killing in the first few hours after challenge. More specifically, the models predict that in mice immunised with the LV there was a substantial reduction (by 44%) in the number of viable bacteria compared to naive mice. This is predicted to occur before the bacteria can be detected in the spleen and liver. The models predict that once the bacteria are in the spleen and liver of the LV-immunised mice they are subject to enhanced bactericidal activity in the spleen and normal bactericidal activity in the liver as compared to naïve mice. For the KV, in contrast, we did not observe any loss of viable bacteria by 30 min p.i., there was then more intense bactericidal activity (combined with faster bacterial replication) in the liver than any other group. Between 6 and 24 h p.i. bacterial replication and killing rates decreased markedly in animals immunized with either vaccine type showing that the control of bacterial numbers after 6 h proceeds mainly by bacteriostatic mechanisms.

Taken together the model estimates, measures of WITS co-occurrence, and correlation between the organs show that immunisation with KV enhances blood clearance, and increases the killing rates in the organs (up to 24 h). Subsequently bacteriostatic effects predominate, but these are not enhanced by the killed vaccine over what is seen in naive animals, as shown by similar increases over time in bacterial numbers in naive and KV mice. In contrast, the immune response induced by the LV rapidly inactivated (within 30 min) a large fraction of the challenge dose, enhanced clearance of the bacteria from the blood and their transfer into the organs and subsequently exerted a stronger bacteriostatic effect which restrained bacterial growth more than in naive or KV animals.

### T-cells do not contribute to early LV-induced restraint of bacterial growth but are important for late bacterial killing

Animals immunised with a live vaccine can control the growth of a secondary challenge in the spleen and liver. CD4^+^ and CD8^+^ T-cells are known to play a key role in immunity conferred by live vaccination [8], [33]. We therefore wished to determine how and at which time T-cell dependent immune functions impact on the dynamics of a secondary challenge in terms of control of bacterial division, enhancement of death and restraint of spread between systemic sites. LV-immunised mice were treated with either anti-CD4 plus anti-CD8 antibodies, or with control immunoglobulins two days before and after challenge with ∼300 CFU of WITS. Pilot experiments showed that differences in bacterial loads between T-cell positive and T-cell negative animals became significant only by 72 h post-challenge therefore we extended the time-course of the experiments to capture these differences; groups of mice (n = 5 or 10, see **Table S1**) were sacrificed at 30 min, 6, 24, 48, 72, 96, 120 and 144 h and total bacterial numbers and WITS population structures were assessed in the spleen, liver and blood.

**Figure 5:**
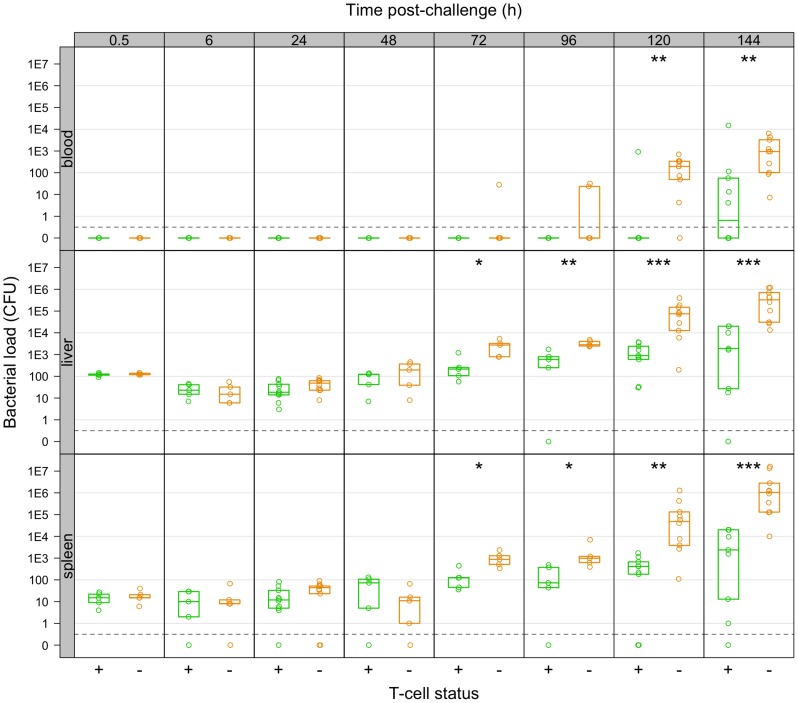
Net growth of secondary challenge bacteria in LV-immunised mice depleted of T-cells as compared to controls. T-cell depleted mice fail to restrain growth of secondary challenge bacteria from 72 h p.i. Bacterial numbers (CFU) in the organs of live-vaccine immunised mice at the indicated times post-challenge with 310 CFU WITS. Mice were controls (T+: green) or depleted of T-cells (T−:red). Dots show values in each mouse, boxes show the quartiles for each group (25%, median and 75%). Significant differences in bacterial loads between T+ and T− mice are shown by: *P<0.05; **P<0.01; ***P<0.001 (Mann-Whitney *U*-test).

Depletion of T-cells had no effect on the bacterial loads of the challenge organisms up to 48 h, but subsequently the groups diverged with the LV immunised T-cell depleted animals having higher loads and from 96 h bacterial numbers in this group were increasing at a rate similar to that seen in naive animal (**Figure 5**). Subsequently bacterial loads in T-cell depleted mice were consistently higher than in LV-immunised control animals and a resurgence of bacteraemia was detected in the T-cell depleted mice. Bacterial counts in the control mice immunised with the LV increased more slowly than in LV-immunised T-cell depleted mice.

**Figure 6:**
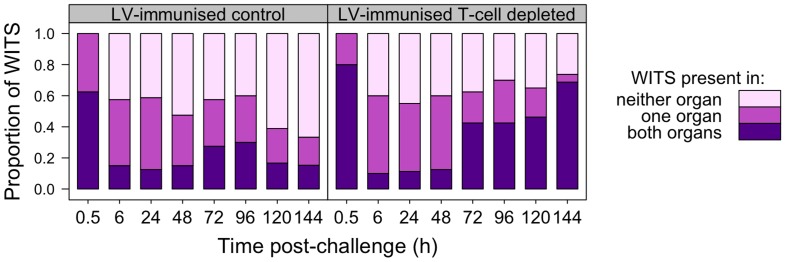
Co-occurrence of WITS in the livers and spleens during secondary challenge. T-cell dependent bactericidal activity is evident in LV-immunised mice by 120 h post-rechallenge; depletion of T-cells from LV-immunised animals results in inter-organ spread of bacteria from 72 h. Each bar shows the distribution of all WITS in each experimental group at the indicated time post-challenge. From bottom up: proportion of WITS present in both liver and spleen (purple); in one organ only (dark pink); in neither organ (light pink).

**Figure 7:**
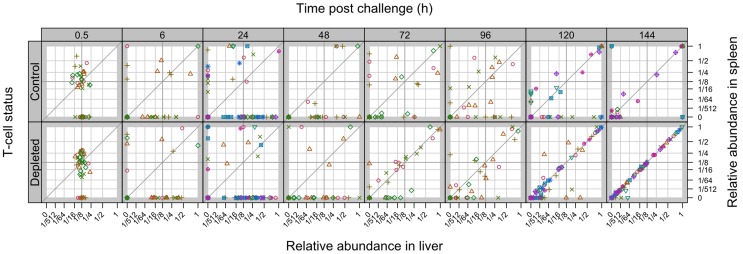
Relative abundance of individual WITS in the spleens and livers. Secondary challenge bacteria segregate into the liver and spleen to form independent populations within 6 h. Subsequently hepatic and splenic populations become homogeneous from 120 h. All WITS within a mouse are represented by the same symbol. Points along the diagonal correspond to equal abundances in both organs. Mice were either controls or depleted of T-cells (T−); they all received the live vaccine and were challenged with 310 CFU of WITS.

Observation of WITS co-occurrence in the spleens and livers (**Figure 6**) showed similar overall bactericidal activity (absence of individual WITS from both organs) up to 96 h post-challenge in both control and T-cell depleted mice. Fitting the mathematical model to this dataset showed that while estimated killing and replication rates over the first 6 h were somewhat higher than in the previously conducted LV experiment, there was little difference between the T-cell depleted and control (T-cell positive) mice (see Supporting Information **Text S1**). By 120 h, the number of distinct WITS in the organs decreased in LV immunised control mice, indicating killing of bacteria had started to occur in LV. Conversely there was no evidence of bacterial killing in T-cell depleted animals and the number of WITS simultaneously present in both organs increased markedly from 72 h post-challenge which was also indicative of inter-organ spread. The relative proportions of individual WITS in this group were similar in the spleens and livers of T-cell depleted mice (**Figure 7** **;** **Table 3**) confirming that inter-organ spread was significant in these animals. A resurgence of bacteraemia was observed at later time-points in vaccinated T-cell depleted animals, this could not have been due to a defect in production of antibody following T-cell depletion as there was no difference in circulating Ig levels between T+ and T− animals post-secondary challenge (**Figure S1**). Unfortunately it was not possible at this time to quantify these processes using our mathematical model due to the increased complexity of the system at later time points.

**Table 3:** Increase in correlation between organ bacterial populations over time.

	% positive correlation	
Time p.i. (h)	T−	T+
0.5	0	0
6	20	20
24	10	10
48	20	40
72	100	40
96	100	20
120	90	67
144	100	75

Percentage of T-cell depleted (T−) or T-cell positive (T+) live-vaccine immunised mice exhibiting a statistically significant positive correlation between the abundances of the WITS in the liver and spleen. This was assessed by computing 95% confidence intervals for the coefficients of correlation (Figure S8). Mice with bacteria absent from both liver and spleen were excluded from the analysis.

We also observed highly correlated WITS organ populations in the majority of LV-immunised T-cell positive mice later in the infection, but only from 120 h (**Table 3**) indicating that in these mice bacteria were able to spread between organs despite the very low-grade bacteraemia. By considering mice individually we see that highly correlated populations were observed in those animals with higher bacterial loads and that only one WITS made up the majority of the population in these mice (**Figure S2**). T-cell depleted animals also showed a limited number of WITS predominating at 144 h although more individual WITS were present in each sample presumably as bacterial killing was not significant in these animals. KV-immunised mice showed the same effect by 72 h post-challenge, as did naive mice albeit to a more limited extent.

Taken together these data show that neither the LV-induced initial rapid bacterial kill nor the subsequent bacteriostatic mechanisms that predominate early in the infection were T-cell dependent and that T-cell dependent bacterial killing became observable by 120 h p.i.

## Discussion

Here we show that previous immunisation with a live attenuated *Salmonella* vaccine results in the rapid kill of a great proportion of the challenge inoculum and additionally enhanced bacteriostasis until the robust LV-induced T-cell response controls the infection by bactericidal mechanisms. LV also controls secondary bacteraemia to extremely low levels and delays and reduces inter-organ spread. Immunisation with a whole-cell killed vaccine enhanced blood clearance and only transiently (within the first 24 h after challenge) increased the rates of bacterial killing within the organs. Later in the infection, the KV neither controlled the net growth of the virulent challenge in the spleen and liver nor was able to induce clearance of the bacteria from the tissues. The KV did not control the rapid resurgence of bacteraemia and did not control inter-organ spread despite its ability to induce anti-*Salmonella* antibodies.

Mixed infections with individually identifiable wild-type isogenic tagged strains (WITS) have recently been used to gather information on the population dynamics of bacteria within hosts [24]–[25], [34]. Use of multiple isogenic strains facilitates more refined measurement of subpopulations than using a more limited number of strains selectable with different antibiotics [35]–[37] as well as limiting the possibility of phenotypic variation between competing strains with different genotypes.

In this study we used WITS to study the *in vivo* population dynamics of *S.* Typhimurium during secondary challenge following vaccination. In the early phase of the challenge (up to 24 h) we estimated bacterial replication and killing rates by mathematical modelling. For later stages in the infection we could infer the times at which inter-organ spread and bacterial killing became observable by tracking the presence or absence of WITS in the organs and calculating the correlation between the abundances of WITS in the organs. We chose the parenteral route to investigate the secondary challenge dynamics in mice immunised with either a killed vaccine or a live attenuated strain. This route enabled us to study specifically the dynamics that underpin control of the bacteria in the systemic compartment. Systemic control of the infection - in other words the suppression of bacterial growth, restraint of dissemination in the spleen, liver and blood, and clearance of the bacteria from the tissues - is an absolute requirement for both host survival in systemic *Salmonella* infections and for the successful elimination of the infection. We used secondary challenge inoculum sizes of ∼300 CFU as the lowest dose that consistently prevented rapid clearance in immunised animals. This dose is similar to a recent estimate of a rate of migration of 298 CFU/day of STm from the gastrointestinal tract into the cecal lymph node following oral infection of mice [34].

We used a host-pathogen combination where a virulent strain was injected as the challenge organism into innately susceptible mice. This stringent combination always results in lethal infections in naïve animals. Bacterial numbers initially decline following challenge due to reactive oxygen radical mediated killing which exceeds division, but subsequently killing becomes negligible and intracellular replication results in a constant and exponential increase in bacterial numbers in the spleen and liver until death of the animal [25]. In this stringent model we showed that the LV induces an immune response that controls a secondary infection in four phases: rapid clearance of bacteria from the blood into the organs coupled with an initial very rapid (within 30 min) inactivation of a large fraction of the challenge dose; a period (∼6 h) of relatively rapid bacterial replication and killing essentially equivalent to that in naive animals; a T-cell independent bacteriostatic phase that lasts for about three days and restrains bacterial growth more than in naive controls; and a subsequent phase in which T-cell dependent bacterial killing becomes significant. Assessment of net bacterial numbers alone would not have distinguished the time of transition between these latter two phases as total bacterial loads were relatively stable over the time period under investigation. The very rapid initial inactivation of the challenge could be a consequence of bacterial killing, or entry into a non-replicative state [21], [31]–[32] that cannot be recovered from following direct plating of organ homogenates. Any early bacterial killing is unlikely to be the result of serum bactericidal activity as mouse serum lacks such activity against *Salmonella* due to a reduced ability to deposit C3 [38]–[39]. We confirmed this experimentally by assessing the survival of *S.* Typhimurium SL1344 in serum collected from mice immunised with the LV (**Figure S3**). Entry into a non-replicative state is also unlikely to account for the large drop in viable bacterial numbers seen as non-replicating salmonellae have not been observed at high levels in the spleen, and resume growth upon culturing [40].

We showed that the whole-cell killed vaccine did not induce the very rapid inactivation of the challenge dose seen in mice immunised with the LV, but did result in a decrease in total bacterial loads as compared to naive controls by 6 h post-challenge. This was primarily due to rapid blood clearance leading to more time being available for bactericidal mechanisms to exert their effect coupled with an increase in killing rate in the liver. While it has been known for many years that living and non-living vaccines can reduce total bacterial loads within the first hours of a re-infection [26]–[30] it is now clear that these reductions proceed with markedly different kinetics. After the first 24 h for animals immunised with the KV there was no additional restraint of bacterial growth, enhancement of killing or control of bacteraemia and the infection proceeded as in naive animals despite the presence of circulating antibodies. We also saw a decrease in the number of WITS absent simultaneously from both livers and spleens at later time-points in KV-immunised animals. This could have been due to re-emergence of bacteria from sites that we did not sample in this study, for example the bone marrow, during the haematogenous spread phase of the infection. This phenomenon was not observed in LV-immunised animals.

Vaccination with live attenuated *Salmonella* is known to induce higher levels of IgG2a (IgG2c in C57BL/6 mice) as compared to a killed vaccine [16], which we also confirmed in our model (**Figure S4**). Opsonisation of *Salmonella* with immune serum increases both the uptake of bacteria by macrophages and bacterial killing *via* the production of reactive oxygen intermediates (ROI) by the phagocyte NADPH oxidase (*phox*) [41]. *phox* also possesses bacteriostatic functions [42], which we showed were important in naive animals *in vivo* after its initial bactericidal effect [25]. Macrophage uptake of IgG-opsonised *Salmonella* is primarily mediated by FcγRI which binds IgG2c [41]. It is therefore possible that both the LV-induced immediate bacterial killing and extended bacteriostasis are due in part to enhanced production of anti-*Salmonella* IgG2c. Reactive nitrogen intermediates (RNI), produced by *iNOS*, are also known to have bacteriostatic activity both *in vitro* and *in vivo* [43]–[44] and while opsonisation has not been reported to increase RNI production by macrophages *in vitro* [41] it is possible that such an effect does occur *in vivo*.

In this study we observed that the bacterial population structures in naive mice, and in KV and LV-immunised mice eventually become homogeneous between the livers and spleens indicating that inter-organ mixing of WITS occurs during the infection process. However, mixing occurs earlier in naive mice, in mice immunised with the KV and in LV-immunised T-cell depleted animals compared to control LV-immunised wild-type mice. This inter-organ mixing coincided with bacteria becoming detectable in the blood suggesting that haematogenous spread is responsible for transfer of bacteria between organs. In the majority of the LV-immunised mice inter-organ mixing was seen in the absence of detectable bacteraemia which leads us to speculate that in these animals very few bacteria are released into the blood at any time due to a more efficient control of bacterial release from the infection foci.

In the KV-vaccinated mice, as the secondary infection progressed a small number of individual WITS predominated within the overall population structure. This phenomenon was also observed in naive control mice in this study although to a lesser extent than was previously seen when naive animals were challenged with a lower dose (∼90 CFU) [45]. Given that we do not observe bacterial killing during the net growth phase of WITS in the tissues in these mice (from 24 h p.i. onwards), the increased prevalence of some WITS is likely to be due to a relatively faster expansion of some WITS populations over others within each organ. *Salmonella* adapt *in vivo* to enter a state in which net intra-organ growth is faster [10] and individual foci of infection arise from distinct clonal populations [46]. We speculate that the expansion of a limited number of WITS populations is a consequence of a loss of control of the infection at a small number of foci (each of which contains a single WITS) resulting in release of bacteria that then establish secondary foci with rapidly growing bacteria [9], [25], [47]–[48].

A similar prevalence of a low number of distinct WITS was also seen in the later stages of the secondary infection in mice immunised with LV suggesting that loss of focal control at a limited number of sites also occurs in these animals. However in these mice we observed a T-cell dependent bactericidal activity of the immune system from 120 h onwards and therefore it is possible that bacterial killing is also responsible for the stochastic prevalence of some bacterial subpopulations over others.

Overall our study indicates the superiority of a live attenuated vaccine over a whole-cell killed one in controlling the infection process by suppressing bacterial growth, exerting bacterial killing and achieving clearance of the inoculum from the tissues. The latter function is ascribable to the T-cell response induced by the LV since it can be abrogated by depletion of T-cells before challenge. This work indicates that a KV is inadequate to control a secondary infection in a very stringent host pathogen combination. New generations of *Salmonella* vaccines such as polysaccharide and subunit vaccines for typhoid and NTS are currently being considered and empirically tested. These preparations rely on the induction of antibodies for their protective activity so the optimisation of these vaccines and their delivery would benefit from improvements in their ability to induce T-cell mediated immunity.

## Materials and Methods

### Bacterial strains, media and growth conditions

*S. enterica* serovar Typhimurium (STm) WITS strains 1, 2, 11, 13, 17, 19, 20 and 21 which have been described previously [25] were made by inserting 40 bp signature tags and a kanamycin resistance cassette between the *malXY* pseudogenes of STm JH3016 [49], a *gfp* ^+^ derivative of wild-type virulent SL1344. They are phenotypically wild-type for growth in broth and infectivity for mice. The live attenuated STm SL3261 *aroA* [50] strain was used for all immunizations. Bacteria for infection were grown for 16 h at 37°C in L-broth (LB) without aeration and diluted in phosphate-buffered saline (PBS) prior to inoculation. Enumeration of bacteria was by plating dilutions on LB agar plates.

### Ethics statement

All experiments involving animals were conducted under project licences approved by the University of Cambridge Animal Welfare and Ethical Review Body, granted by the United Kingdom Home Office (licence numbers PPL 80/2135 and PPL 80/2572), and performed in observance of licensed procedures under the United Kingdom Animals (Scientific Procedures) Act 1986.

### Immunization and infections

Female age-matched C57BL/6 mice were purchase from Harlan Laboratories and used when over 9 weeks of age. Live bacteria for parenteral immunization were prepared from a 16 hr static culture of STm SL3261, diluted 1/100 in PBS and administered by *i.v.* injection into the tail vein in 200 µl aliquots (∼10^6^ CFU/mouse). Actual inoculum dose was determined by plating dilutions. We confirmed that this immunisation regime elicited anti-*Salmonella* antibodies (**Figure S1**) and elicited a T_H_1-type memory response (**Figure S5**). Challenge with WITS was performed three months after immunisation with the LV by which time the primary infection was cleared, as determined by sampling livers and spleens of mice in pilot experiments (*n* = 2), and demonstrated previously [51].

Acetone killed wild-type bacteria for immunization were prepared as previously described [16]. Briefly, bacteria from a 100 ml culture of STm SL1344 grown for 16 h at 37°C with aeration in tryptic soy broth (Oxoid) were harvested by centrifugation at 3220 *g*, resuspended in 100 ml PBS and viable counts determined by plating. Following three sequential washes in acetone, bacteria were harvested, acetone completely removed by evaporation, cells resuspended in PBS to an equivalent concentration of 5×10^10^ CFU/ml and stored in aliquots at −20°C or below. Immunization was by administration of two doses of 10^8^ cells given subcutaneously in the back, 3-weeks apart. The subcutaneous route was chosen as in our experience this results in a strong antibody response with the killed vaccine whereas *i.v.* immunisation does not. Regardless of route the KV is known to be unable to induce protective T-cell immunity against virulent challenge. We confirmed that this immunisation regime elicited anti-*Salmonella* antibody, although there was a somewhat lower production of IgG as compared to immunisation with the LV, due to a decrease in levels of IgG2c (**Figure S4**). Challenge with WITS was performed 6 weeks post-primary immunisation.

For T-cell depletion experiments 500 µg each of rat IgG2b anti-mouse CD4 and anti-mouse CD8 (prepared by Harlan Bioproducts from hybridomas YTS 191.1 and YTS 169.4.2 respectively. Hybridoma lines were a kind gift of Prof. Anne Cooke, University of Cambridge) in PBS were injected into the tail vein at days −2 and +2 with respect to the challenge [33], [52]–[53]. Control animals received normal rat globulins (mpBiomedical). In this model the development of immunity post-vaccination proceeds normally as the mice are wild-type; removal of T-cells only occurs around the time of challenge. This regime was confirmed by FACS to deplete the mice of both CD4^+^ and CD8^+^ splenocytes (**Figure S6**).

For infections with WITS, each strain was individually grown statically for 16 h in L-broth and aliquots mixed to obtain a stock with each strain in an equal amount. Bacteria from 1 ml of this stock were harvested by centrifugation for subsequent qPCR. Dilutions of the stock were then made in PBS (typically 1 in 5×10^5^) to achieve the desired final cell density for *i.v.* injection of 200 µl doses into the tail vein. Actual cell density of the inoculums were determined by plating triplicate 200 µl aliquots, cell density in the individual WITS cultures was also determined. Experimental group sizes are shown in **Table S1**.

To enable mathematical modelling of subpopulation structures we attempted to determine a single challenge dose that would result both in consistent colonisation of the organs in immunised animals, and a frequency of WITS absence in naive animals that would enable paramaterisation of our previously developed model; in naive animals a dose of ∼90 CFU is appropriate [25]. We conducted pilot experiments where animals immunised with the LV or naive controls were challenged with 90, 300, 900 or 9000 CFU total WITS and we determined the WITS population structure in spleens and livers at 6 h post-infection (**Table S2**) when bacterial counts are at a minimum in naive animals [25]. At the lowest dose there was excessive WITS loss in the spleen of immunised animals while at the other doses there was insufficient loss of WITS from naive animals. We therefore selected a challenge dose of ∼300 CFU which consistently resulted in colonisation of the spleen in immunised animals and modified the mathematical models to account for variations in the relative proportions of WITS between animals, rather than merely for the presence or absence of subpopulations.

### Enumeration and recovery of viable *Salmonella* in blood and tissues

Blood was obtained from the tail vein in heparin-coated tubes, mice were humanely killed by cervical dislocation, spleens and livers removed and individually homogenised in a Stomacher80 (Seward) with 5 ml distilled water. If required, dilutions were made to enable enumeration by pour plating 100 µl aliquots in 6-well plates. Entire blood samples or tissue homogenates in 1 ml aliquots were inoculated onto the surface of 90 mm agar plates. Following overnight incubation at 37°C, colonies were enumerated and total bacteria harvested from the plates by washing with 2 ml PBS. Bacteria were thoroughly mixed by vortexing, harvested by centrifugation and stored at −80°C prior to DNA extraction.

### Determination of WITS proportions in bacterial samples by qPCR

DNA was prepared from aliquots of bacterial samples (typically ∼5×10^9^ CFU) using a DNeasy Blood and Tissue kit (QIAGEN). DNA concentration was determined using a NanoDrop 1000 spectrophotometer (Thermo Scientific). Approximately 10^6^ total genome copies were analysed for the relative proportions of each WITS by qPCR on a Rotor-Gene Q (QIAGEN). Duplicate reactions were performed for each sample with primer pairs specific for each WITS in separate 20 µl reactions (primers in **Table S3**). Each reaction contained 10 µl QuantiTect SYBR Green (QIAGEN), 1 µM each primer, 4 µl sample and DNase-free water to 20 µl. Thermal cycling was 95°C for 15 min; 35 cycles of 94°C for 15 s, 61°C for 30 s, and 72°C for 20 s. The copy number of each WITS genome in the sample was determined by reference to standard curves for each primer pair. Standard curves were generated for each batch of PCR reagents by performing qPCRs in duplicate on 4 separate dilution series of known concentrations of WITS genomic DNA.

### Mathematical model and statistical inference

We used a branching process model that kept track of the distribution of the number of bacteria of a single WITS in three compartments: blood, liver and spleen. The model is outlined here, a full description is in Supporting Information **Text S1**. While we had determined the average number of CFU in the inoculum by plating, total bacterial loads in the LV immunized animals at 30 min were markedly lower, presumably due to the rapid activity of bactericidal mechanisms. In order to account for this observation we assumed that at time *t* = 0, the bacteria followed a Poisson distribution with unknown mean ν in the blood, whence they migrate into the liver (at rate *c_L_*) and the spleen (at rate *c_S_*). Upon entering the liver, bacteria replicate at rate *r_L_* and are killed at rate *k_L_*. Likewise, those in the spleen replicate at rate *r_S_* and are killed at rate *k_S_*. For the sake of parsimony, we assume that these rates remain constant for the first six hours, and we estimate them by fitting the model to the data from first two time points (0.5 h and 6 h post inoculation). We then allow the replication and killing rates to change at *t* = 6 h and assume they remain constant until *t* = 24 h; we estimate these new values by fitting the model to the data from the third time point (24 h p.i.). For each experimental treatment, we thus estimate 11 parameters (*ν*, *c_L_*, *c_S_*, *k_L1_*, *k_S1_*, *r_L1_*, *r_S1_*, *k_L2_*, *k_S2_*, *r_L2_*, *r_S2_*) from up to 720 data points (3 time points ×9 or 10 mice ×3 compartments ×8 strains), where subscripts _1_ and _2_ refer to the two time intervals considered (0–6 h and 6–24 h respectively) We assumed that all strains and all mice were independent and shared identical parameters within each experimental group. Our model did not allow movement of bacteria from the liver or the spleen back into the blood, as data strongly suggested that this does not happen until after 24 h [25].

Inference was done by maximum likelihood; the complete mathematical formulation is presented in Supporting Information **Text S1**. Basically, for a given set of model parameters, we computed the joint probability distribution of the number of copies of a single WITS in the blood, liver and spleen during the first 24 h by solving the master equations of the branching process. In order to compute the likelihood of the model, we also needed to determine the probability of observing a set of data (CFU and proportions of the 8 WITS in the blood, liver and spleen from a given mouse) given the unobserved numbers of copies of each WITS that were actually present in the blood and organs of that mouse (which correspond to the variables in our stochastic model); in other words, we had to estimate the noise generated by the experimental procedure. This was achieved by an additional calibration experiment: we plated known numbers of each WITS, harvested the colonies and processed known combinations of the eight WITS by qPCR. We then performed regressions of the observed proportions of the WITS by qPCR against the known numbers of colonies. We compared six models by AIC (Akaike's Information Criterion) for the distribution of *ω* (the product of the total number of colonies harvested by the proportion of a single WITS reported by qPCR) given *n* (the actual number of colonies of this particular WITS on the plate). The most parsimonious was a log-normal distribution where the log-standard-deviation decreases exponentially with *n*, namely: log(*ω*)∼N(log(*n*),0.267e^−0.0148*n*^). See Supporting Information **Text S1** for further detail. All the analyses were performed in R version 3.0 [54], and likelihood estimation was done using the R library Powell [55].

## Supporting Information

Figure S1 **Anti-** ***Salmonella*** **antibody response following immunisation with live vaccine.**
(PDF)

Figure S2 **Individual WITS present in livers and spleens, ordered by total bacterial load in the animals.**
(PDF)

Figure S3 **Serum from LV-immunised mice does not possess bactericidal activity.**
(PDF)

Figure S4 **Anti-** ***Salmonella*** **antibody isotype and IgG subclass response following immunisation.**
(PDF)

Figure S5 **Cytokine production by** ***Salmonella*** **-antigen stimulated CD4^+^ T-cells shows induction of a T_H_1 memory response.**
(PDF)

Figure S6 **FACS analaysis showing depletion of T-cells.**
(PDF)

Figure S7 **Hepatic and splenic WITS population correlation coefficients for LV and KV groups.**
(PDF)

Figure S8 **Hepatic and splenic WITS population correlation coefficients for LV-immunised, T-cell positive and T-cell negative groups.**
(PDF)

Table S1 **Experimental group sizes.**
(PDF)

Table S2 **Presence and absence of WITS in LV-immunised and naive mice at various secondary challenge doses.**
(PDF)

Table S3 **Primers used for qPCR.**
(PDF)

Text S1 **Supplementary methods and results—stochastic model and inference.**
(PDF)

## References

[1] Crump JA, Mintz ED (2010) Global trends in typhoid and paratyphoid fever. Clin Infect Dis 50: 241–246. 20014951 10.1086/649541 PMC2798017

[2] Gordon MA (2011) Invasive non-typhoidal *Salmonella* disease - epidemiology, pathogenesis and diagnosis. Curr Opin Infect Dis 24: 484–489. 21844803 10.1097/QCO.0b013e32834a9980 PMC3277940

[3] Layton AN, Galyov EE (2007) *Salmonella*-induced enteritis: molecular pathogenesis and therapeutic implications. Expert Rev Mol Med 9: 1–17. 10.1017/S1462399407000373 17605831

[4] Mulholland EK, Adegbola RA (2005) Bacterial infections - a major cause of death among children in Africa. New Engl J Med 352: 75–77. 15635117 10.1056/NEJMe048306

[5] Hohmann EL (2001) Nontyphoidal salmonellosis. Clin Infect Dis 32: 263–269. 11170916 10.1086/318457

[6] Parry CM, Threlfall EJ (2008) Antimircobial resistance in typhoidal and nontyphoidal salmonellae. Curr Opin Infect Dis 21: 531–538. 18725804 10.1097/QCO.0b013e32830f453a

[7] McGregor AC, Waddington CS, Pollard AJ (2013) Prospects for prevention of *Salmonella* infection in children through vaccination. Curr Opin Infect Dis 26: 254–262. 23591641 10.1097/QCO.0b013e32835fb829

[8] Mastroeni P, Villareal-Ramos B, Hormaeche CE (1993) Adoptive transfer of immunity to oral challenge with virulent salmonellae in innately susceptible BALB/c mice requires both immune serum and T cells. Infect Immun 61: 3981–3984. 8359920 10.1128/iai.61.9.3981-3984.1993 PMC281103

[9] Mastroeni P, Grant A, Restif O, Maskell D (2009) A dynamic view of the spread and intracellular distribution of *Salmonella enterica* . Nat Rev Microbiol 7: 73–80. 19079353 10.1038/nrmicro2034

[10] Mastroeni P, Morgan FJE, McKinley TJ, Shawcroft E, Clare S, et al (2011) Enhanced virulence of *Salmonella enterica* serovar Typhimurium after passage through mice. Infect Immun 79: 636–643. 21098099 10.1128/IAI.00954-10 PMC3028859

[11] Guzman CA, Borsutsky S, Griot-Wenk M, Metcalfe IC, Pearman J, et al (2006) Vaccines against typhoid fever. Vaccine 24: 3804–3811. 16278037 10.1016/j.vaccine.2005.07.111

[12] Sztein MB (2007) Cell-mediated immunity and antibody responses elicited by attenuated *Salmonella enterica* erovar Typhi strains used as live oral vaccines in humans. Clin Infect Dis 45: S15–S19. 17582562 10.1086/518140

[13] Szu SC (2013) Development of Vi conjugate - a new generation of typhoid vaccine. Expert Review of Vaccines 12: 1273–1286. 24156285 10.1586/14760584.2013.845529

[14] van Damme P, Kafeja F, Anemona A, Basile V, Hilbert AK, et al (2011) Safety, Immunogenicity and Dose Ranging of a New Vi-CRM_197_ Conjugate Vaccine against Typhoid Fever: Randomized Clinical Testing in Healthy Adults. PLoS One 6: e25398. 21980445 10.1371/journal.pone.0025398 PMC3184126

[15] Eisenstein TK, Killar LM, Sultzer BM (1984) Immunity to infection with *Salmonella typhimurium*: mouse-strain differences in vaccine- and serum-mediated protection. J Infect Dis 150: 425–435. 6207248 10.1093/infdis/150.3.425

[16] Harrison JA, Villareal-Ramos B, Mastroeni P, De Hormaeche RD, Hormaeche CE (1997) Correlates of protection induced by live Aro^-^ *Salmonella typhimurium* vaccines in the murine typhoid model. Immunology 90: 618–625. 9176117 10.1046/j.1365-2567.1997.00158.x PMC1456680

[17] Levine MM, Ferreccio C, Black RE, Tacket CO, Germanier R (1989) Progress in vaccines against typhoid fever. Rev Infect Dis 11 Suppl 3. 10.1093/clinids/11.supplement_3.s552 2669099

[18] Mastroeni P, Chabalgoity JA, Dunstan SJ, Maskell DJ, Dougan G (2001) *Salmonella*: immune responses and vaccines. The Vet J 161: 132–164. 11243685 10.1053/tvjl.2000.0502

[19] Thatte J, Rath S, Bal V (1993) Immunization with live versus killed *Salmonella typhimurium* leads to the generation of an IFN-gamma-dominant versus an IL-4-dominant immune response. International Immunology 5: 1431–1436. 8260457 10.1093/intimm/5.11.1431

[20] Benjamin WH Jr, Hall P, Roberts SJ, Briles DE (1990) The primary effect of the *Ity* locus is on the rate of growth of *Salmonella typhimurium* that are relatively protected from killing. J Immunol 144: 3143–3151. 2182715

[21] Helaine S, Holden DW (2013) Heterogeneity of intracellular replication of bacterial pathogens. Curr Opp Microbiol 16: 184–191. 10.1016/j.mib.2012.12.004 23485258

[22] Hormaeche CE (1980) The *in vivo* division and death rates of *Salmonella typhimurium* in the spleens of naturally resistant and susceptible mice measured by the superinfecting phage technique of Meynell. Immunology 41: 973–979. 7007218 PMC1458291

[23] Smith H (2000) Questions about the behavious of bacterial pathogens *in vivo* . Phil Trans R Soc B 255: 551–564. 10.1098/rstb.2000.0597 PMC1692770 10874729

[24] Coward C, van Diemen PM, Conlan AJ, Gog JR, Stevens MP, et al (2008) Competing isogenic *Campylobacter* strains exhibit variable population structures *in vivo* . Appl Environ Microbiol 74: 3857–3867. 18424530 10.1128/AEM.02835-07 PMC2446568

[25] Grant AJ, Restif O, McKinley TJ, Sheppard M, Maskell DJ, et al (2008) Modelling within-host spatiotemporal dynamics of invasive bacterial disease. PLoS Biol 6: e74. 18399718 10.1371/journal.pbio.0060074 PMC2288627

[26] Blanden RV, Mackaness GB, Collins FM (1966) Mechanisms of acquired resistance in mouse typhoid. J Exp Med 124: 585–600. 4958757 10.1084/jem.124.4.585 PMC2138250

[27] Collins FM (1969) Effect of specific immune mouse serum on the growth of *Salmonella enteritidis* in mice preimmunized with living or ethyl alcohol-killed vaccines. J Bacteriol 97: 676–683. 5773021 10.1128/jb.97.2.676-683.1969 PMC249745

[28] Collins FM, Mackaness GB, Blanden RV (1966) Infection-immunity in experimental salmonellosis. J Exp Med 124: 601–619. 5922286 10.1084/jem.124.4.601 PMC2138251

[29] Collins FM, Milne M (1966) Heat-labile antigens of *Salmonella enteritidis* . J Bacteriol 92: 549–557. 5922532 10.1128/jb.92.3.549-557.1966 PMC276288

[30] Mackaness GB, Blanden RV, Collins FM (1966) Host-parasite relations in mouse typhoid. J Exp Med 124: 573–583. 5922285 10.1084/jem.124.4.573 PMC2138243

[31] Helaine S, Thompson JA, Watson KG, Liu M, Boyle C, et al (2010) Dynamics of intracellular bacterial replication at the single cell level. P Natl Acad Sci USA 107: 3746–2751. 10.1073/pnas.1000041107 PMC2840444 20133586

[32] Restif O, Goh YS, Palayret M, Grant AJ, McKinley TJ, et al (2012) Quantification of the effects of antibodies on the extra- and intracellular dyanamics of *Salmonella enterica* . J R Soc Interface 10: 20120866. 23235264 10.1098/rsif.2012.0866 PMC3565705

[33] Mastroeni P, Villareal-Ramos B, Hormaeche CE (1992) Role of T cells, TNFα and IFNγ in recall of immunity to oral challenge with virulent salmonellae in mice vaccinated with live attenuated *aro* ^−^ salmonella vaccines. Microb Pathogenesis 13: 477–491. 10.1016/0882-4010(92)90014-f 1363824

[34] Kaiser P, Slack E, Grant AJ, Hardt W-D, Regoes RR (2013) Lymph node colonization dynamics after oral *Salmonella* Typhimurium infection in mice. PLoS Pathog 9: e1002532. 10.1371/journal.ppat.1003532 PMC3777876 24068916

[35] Meynell GG (1957) The applicability of the hypothesis of independent action to fatal infections in mice given *Salmonella typhimurium* by mouth. J Gen Microbiol 16: 396–404. 13416517 10.1099/00221287-16-2-396

[36] Meynell GG, Stocker BAD (1957) Some hypotheses on the aetiology of fatal infections in partially resistant hosts and their application to mice challenged with *Salmonella paratyphi-B* or *Salmonella typhimurium* by intraperitoneal injection. J Gen Microbiol 16: 38–58. 13406218 10.1099/00221287-16-1-38

[37] Moxon ER, Murphy PA (1978) *Haemophilus influenzae* bacteremia and meningitis resulting from survival of a single organism. P Natl Acad Sci USA 75: 1534–1536. 10.1073/pnas.75.3.1534 PMC411507 306628

[38] Marcus S, Esplin DW, Donaldson DM (1954) Lack of bactericidal effect of mouse serum on a number of common microorganisms. Science 119: 877. 13168383 10.1126/science.119.3103.877

[39] Siggins MK, Cunningham AF, Marshall JL, Chamberlain JL, Henderson IR, et al (2011) Absent bactericidal activity of mouse serum against invasive African nontyphoidal *Salmonella* results from impaired complement function but not lack of antibody. J Immunol 186: 2365–2371. 21217014 10.4049/jimmunol.1000284

[40] Helaine S, Cheverton AM, Watson KG, Faure LM, Matthews SA, et al (2014) Internalization of *Salmonella* by macrophages induces formation of nonreplicating persisters. Science 343: 204–208. 24408438 10.1126/science.1244705 PMC6485627

[41] Uppington H, Ménager N, Boross P, Wood J, Sheppard M, et al (2006) Effect of immune serum and role of individual Fcγ receptors on the intracellular distribution and survival of *Salmonella ennterica* serovar Typhimurium in murine macrophages. Immunology 119: 147–158. 16836651 10.1111/j.1365-2567.2006.02416.x PMC1782356

[42] Rosenberger CM, Finlay BB (2002) Macrophages inhibit *Salmonella* Typhimurium replication through MEK/ERK kinase and phagocyte NADPH oxidase activities. J Biol Chem 277: 18753–18762. 11821396 10.1074/jbc.M110649200

[43] Mastroeni P, Vazquez-Torres A, Fang FC, Xu Y, Khan S, et al (2000) Antimicrobial actions of the NADPH phagocyte oxidase and inducible nitric oxide synthase in experimental salmonellosis. II. Effects on microbial proliferation and host survival *in vivo* . J Exp Med 192: 237–247. 10899910 10.1084/jem.192.2.237 PMC2193252

[44] Vazquez-Torres A, Jones-Carson J, Mastroeni P, Ischiropouloa H, Fang FC (2000) Antimicrobial actions of the NADPH phagocyte oxidase and inducible nitric oxide synthase in experimental salmonellosis. I. Effects on microbial killing by activated peritoneal macrophages *in vitro* . J Exp Med 192: 227–236. 10899909 10.1084/jem.192.2.227 PMC2193262

[45] Grant AJ, Sheppard M, Deardon R, Brown SP, Foster G, et al (2008) Caspase-3-dependent phagocyte death during systemic *Salmonella enterica* serovar Typhimurium infection of mice. Immunology 125: 28–37. 18298550 10.1111/j.1365-2567.2008.02814.x PMC2526257

[46] Sheppard M, Webb C, Heath F, Mallows V, Emilianus R, et al (2003) Dynamics of bacterial growth and distribution within the liver during *Salmonella* infection. Cell Microbiol 5: 593–600. 12925129 10.1046/j.1462-5822.2003.00296.x

[47] Mastroeni P, Grant AJ (2011) Spread of *Salmonella enterica* in the body during systemic infection: unravelling host and pathogen determinants. Expert Rev Mol Med 13: e12. 21477411 10.1017/S1462399411001840

[48] Mastroeni P, Grant AJ (2013) Dynamics of spread of *Salmonella enterica* in the systemic compartment. Microbes Infect 15: 849–857. 24183878 10.1016/j.micinf.2013.10.003

[49] Hauteforte I, Proença MJ, Hinton JC (2003) Single-copy green fluorescent protein gene fusions allow accurate measurement of *Salmonella* gene expression *in vitro* and during infection of mammalian cells. Appl Environ Microbiol 69: 7480–7491. 14660401 10.1128/AEM.69.12.7480-7491.2003 PMC310007

[50] Hoiseth SK, Stocker BA (1981) Aromatic-dependent *Salmonella typhimurium* are non-virulent and effective as live vaccines. Nature 291: 238–239. 7015147 10.1038/291238a0

[51] Ménager N, Foster G, Ugrinovic S, Uppington H, Verbeek S, et al (2007) Fcγ receptors are crucial for the expression of acquired resistance to virulent *Salmonella enterica* serovar Typhimurium *in vivo* but are not required for the induction of humoral or T-cell-mediated immunity. Immunology 120: 424–432. 17328787 10.1111/j.1365-2567.2006.02527.x PMC2265895

[52] Cobbold SP, Jayasuriya A, Nash A, D PT, Waldmann H (1984) Therapy with monoclonal antibodies by elimination of T-cell subsets *in vivo* . Nature 312: 548–551. 6150440 10.1038/312548a0

[53] Cobbold SP, Martin G, Quin S, Waldmann H (1986) Monoclonal antibodies to promote marrow engraftment and tissue graft tolerance. Nature 323: 164–166. 3528866 10.1038/323164a0

[54] R Core Team (2013) R: A language and environment for statistical computing. Vienna, Austria: R Foundation for Statistical Computing.

[55] Dorai-Raj S (2006) Powell's UObyQA algorithm.

