# Supplementary material for: The Effects of Vaccination and Immunity on Bacterial Infection Dynamics In Vivo
Source: PLoS Pathog. 2014 Sep 18;10(9):e1004359. doi: 10.1371/journal.ppat.1004359 (PMC4169467; doi:10.1371/journal.ppat.1004359)
Supplement: Figure S1 — Anti- Salmonella antibody response following immunisation with live vaccine. (PDF) [file ppat.1004359.s001.pdf]

Figure S1: Anti-*Salmonella* antibody response following immunisation with live vaccine
To monitor the antibody response following immunisation, total anti-*Salmonella* LPS immunoglobulin was measured by ELISA in serum from mice 3 months after immunisation with live attenuated STm SL3261 (*n*=5); Group LV.

To monitor the antibody response in T-cell depleted animals following secondary challenge, serum was also collected from the following groups 5 days post-secondary challenge with ~300 CFU STm SL1344:
LV T+: LV-immunised control animals (*n*=7)
LV T-: LV-immunised animals depleted of T-cells with anti-CD4 and anti-CD8 antibodies (*n*=5)

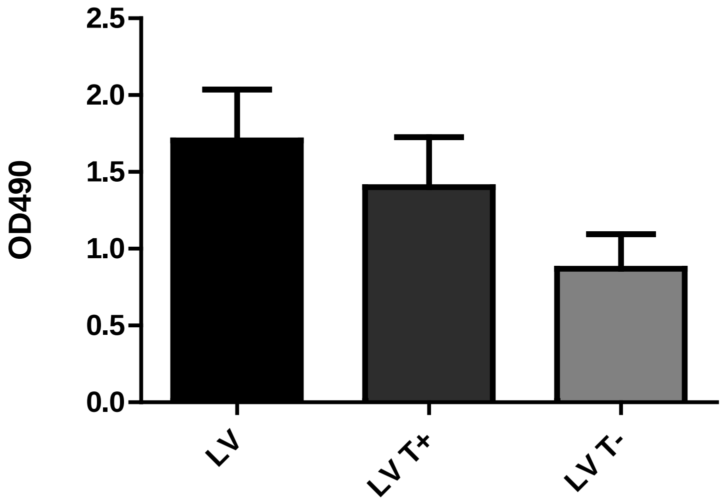

ELISA Method: *Salmonella enterica* serovar Typhimurium LPS (Sigma) was dissolved in water to 1 mg/ml, mixed with an equal volume of sodium deoxycholate (0.5% w/v), incubated at 37 C for 15 min, aliquoted and stored at -20 C. Half-area microtitre plates (Greiner Bio-One) were coated overnight at 37 C with 50 μl LPS at 5 μg/ml in carbonate buffer (0.15% w/v Na_2CO_3; 0.1% w/v $MgCl_2$-$6H_2O$; 0.3% w/v $NaHCO_3$), washed three times with PBS-Tween (0.05%) and blocked with 150 μl PBS+2% BSA for 1 hr at 37 C. Following three further washes, 50 μl serum dilutions (1/5000 in PBS-Tween+1% BSA) were applied in duplicate and incubated for 2 hr at 37 C. Plates were washed and total antibody detected with horseradish peroxidase-conjugated goat anti-mouse Ig antibody (Southern Biotech), detection was with SigmaFast OPD substrate (Sigma). Bars show mean SEM. There was no significant difference between the groups at the 5% level as determined by the Kruskal-Wallis test.
