# Supplementary material for: The Effects of Vaccination and Immunity on Bacterial Infection Dynamics In Vivo
Source: PLoS Pathog. 2014 Sep 18;10(9):e1004359. doi: 10.1371/journal.ppat.1004359 (PMC4169467; doi:10.1371/journal.ppat.1004359)

Figure S2: Individual WITS present in livers and spleens, ordered by total bacterial load in the animals. Hepatic and splenic populations at later timepoints are homogenous and consist of a limited number of distinct WITS. The plots in each panel display the WITS present in individual mice at the indicated time post-challenge (with ~250-300 CFU WITS). Numbers above each plot show the total bacterial load in the animal at this timepoint.

A: LV-immunised, T-cell positive mice at 144 hr post-challenge
B: LV-immunised, T-cell negative mice at 144 hr post-challenge
C: KV-immunised mice at 72 hr post-challenge
D: unimmunised mice at 72 hr post-challenge

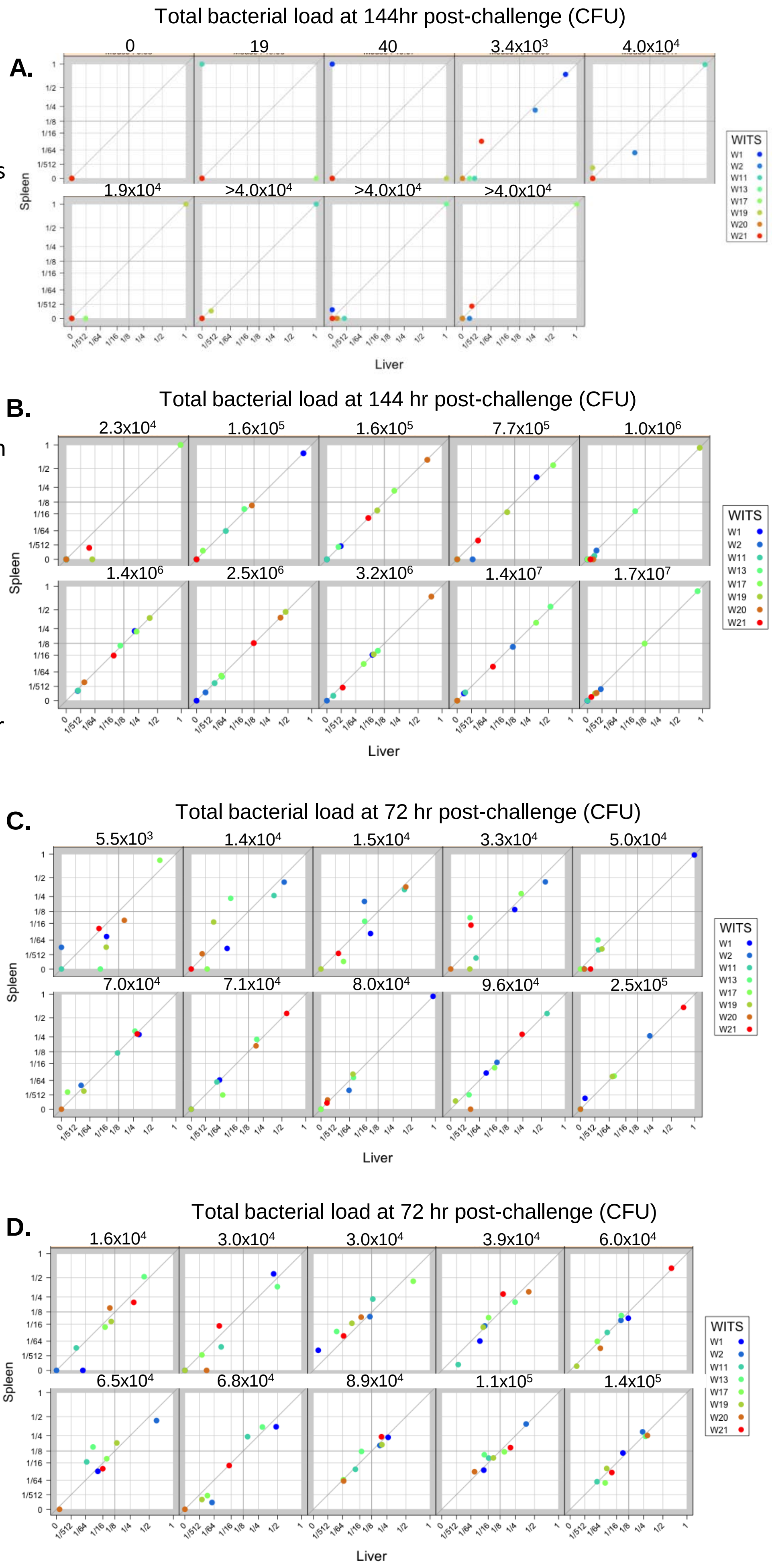

Supplement: Figure S2 — Individual WITS present in livers and spleens, ordered by total bacterial load in the animals. (PDF) [file ppat.1004359.s002.pdf]
