# Supplementary material for: The Effects of Vaccination and Immunity on Bacterial Infection Dynamics In Vivo
Source: PLoS Pathog. 2014 Sep 18;10(9):e1004359. doi: 10.1371/journal.ppat.1004359 (PMC4169467; doi:10.1371/journal.ppat.1004359)

Figure S3: Serum from LV-immunised mice does not possess bactericidal activity.
4 µl of a 1/50 dilution of an overnight culture of STm SL1344 was added to 36 µl immune serum (IMS) or heat-treated serum (HTS) in quadruplicate. After incubation at 37ºC for 30 min, 100 µl of a 1/10^3 dilution was plated onto LB agar and CFU enumerated. Serum was collected from C57BL/6 mice immunised 3 months earlier with ~10^5 CFU live attenuated STm SL3261.

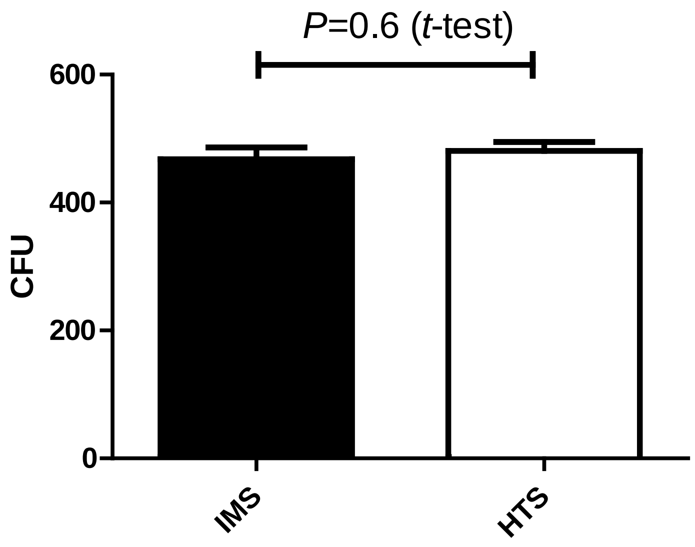

Supplement: Figure S3 — Serum from LV-immunised mice does not possess bactericidal activity. (PDF) [file ppat.1004359.s003.pdf]
