# Supplementary material for: The Effects of Vaccination and Immunity on Bacterial Infection Dynamics In Vivo
Source: PLoS Pathog. 2014 Sep 18;10(9):e1004359. doi: 10.1371/journal.ppat.1004359 (PMC4169467; doi:10.1371/journal.ppat.1004359)
Supplement: Figure S5 — Cytokine production by Salmonella -antigen stimulated CD4+ T-cells shows induction of a TH1 memory response. (PDF) [file ppat.1004359.s005.pdf]

Figure S5: Cytokine production by *Salmonella*-antigen stimulated CD4⁺ T-cells shows induction of a Th1 memory response

Splenocytes were prepared from mice 10 weeks after immunisation with ~10^6 CFU SL3261 or naive controls and enriched for $CD4^+$ T-cells with magnetic beads (Miltenyi Biotech). Cells were then stimulated with *Salmonella* SL1344 alkali-treated whole-cell extract [S1]; PBS (negative control); or anti-CD3 plus anti-CD28 antibody (positive control). Cytokine levels in the supernatant were analysed after 24 hr (IL2) or 72 hr (IFNγ) with DuoSet ELISA kits (R&D Systems)

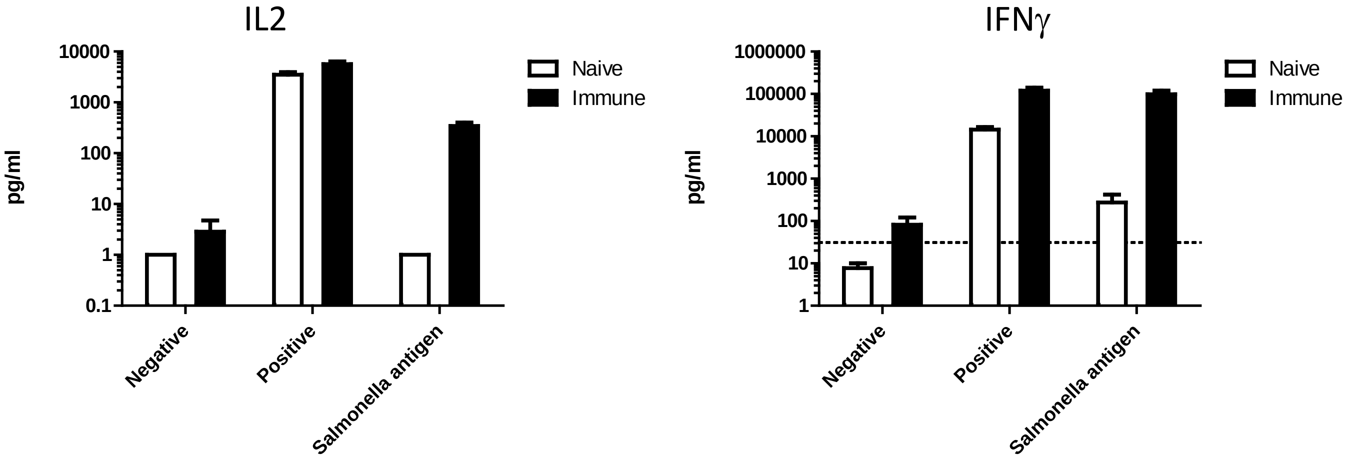

S1. Harrison JA, Villareal-Ramos B, Mastroeni P, De Hormaeche RD, Hormaeche CE (1997) Correlates of protection induced by live Aro⁻ *Salmonella typhimurium* vaccines in the murine typhoid model. Immunology 90: 618-625.
