# Supplementary material for: The Effects of Vaccination and Immunity on Bacterial Infection Dynamics In Vivo
Source: PLoS Pathog. 2014 Sep 18;10(9):e1004359. doi: 10.1371/journal.ppat.1004359 (PMC4169467; doi:10.1371/journal.ppat.1004359)

Fig S6: FACS analysis showing depletion of T-cells.
Splenocytes were prepared from mice treated with either anti-CD4 and anti-CD8 antibodies (Depleted) or control immunoglobulins as described in the methods. FACS was performed on an Accuri C6 (Beckton Dickinson) and data analysed with FlowJo X. Representative plots are shown.

Panel A: CD3+ cells are removed in Depleted group animals
The total T-cell population was determined by staining with anti-mouse CD3e PerCP-eFluor710 (clone 500A2; eBioscience) and comparing to isotype control.

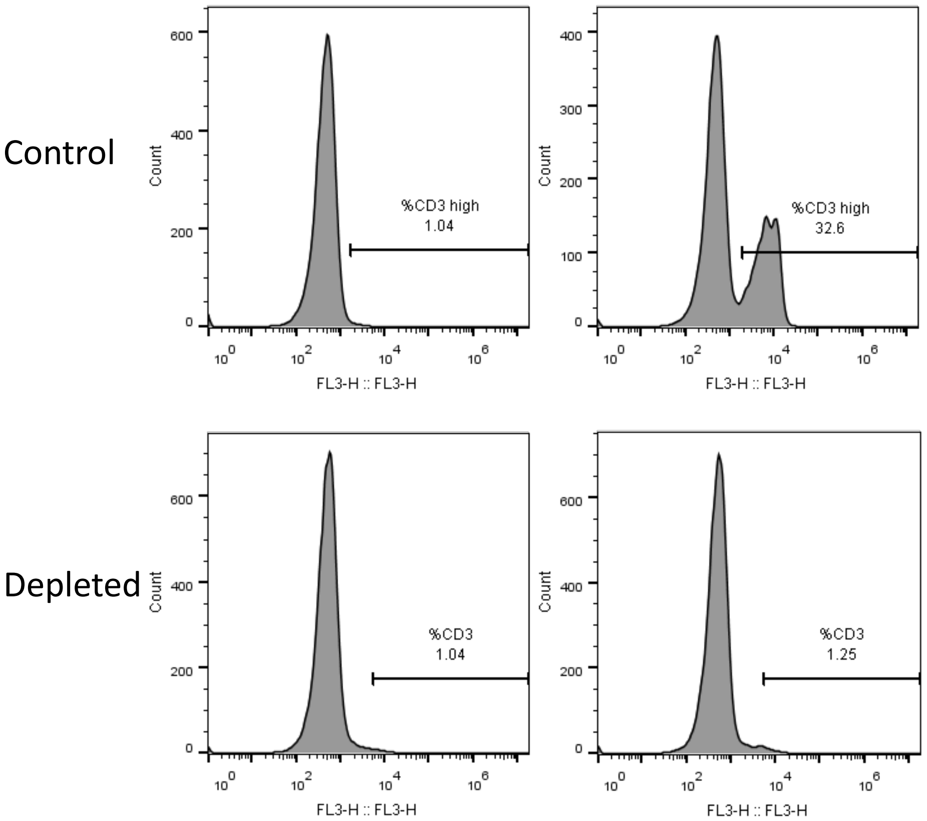

Panel B: Both CD4+ and CD8+ cells are removed in Depleted group animals

The anti-CD4 and anti-CD8 depleting antibodies masked monoclonal detection antibodies during FACS analysis in T-cell depleted animals. Therefore we first incubated splenocytes with the anti-CD4 or anti-CD8 antibodies (both rat IgG2b) or control immunoglobulins, washed and then used anti-rat IgG2b-PE (clone R2B-7C3; eBioscience) as a secondary antibody. In T-cell positive animals the depleting primary antibody will bind and be detected by the secondary whereas in T-cell negative antibodies there will be no binding of the primary and therefore no subsequent detection.

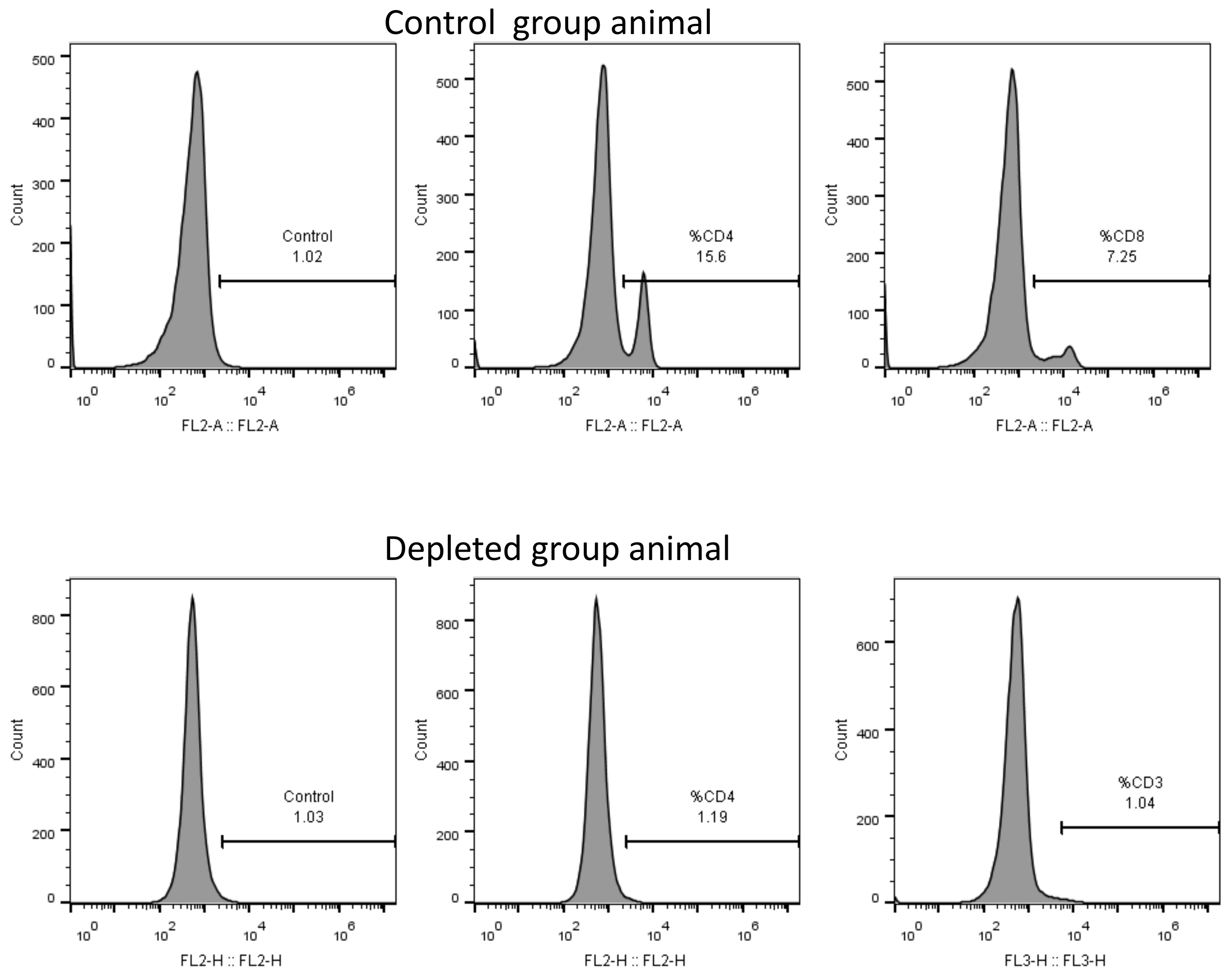

Supplement: Figure S6 — FACS analaysis showing depletion of T-cells. (PDF) [file ppat.1004359.s006.pdf]
