# Supplementary material for: The Effects of Vaccination and Immunity on Bacterial Infection Dynamics In Vivo
Source: PLoS Pathog. 2014 Sep 18;10(9):e1004359. doi: 10.1371/journal.ppat.1004359 (PMC4169467; doi:10.1371/journal.ppat.1004359)

**Figure S8:** Correlation coefficients with 95% confidence intervals for the relative abundance of individual WITS between liver and spleen in live-vaccine immunised mice depleted of T-cells or controls at the indicated times (hr) post-challenge; each dot represents a single mouse. Highlighted in red are those mice with a confidence interval that does not include zero. Animals with bacteria absent from the spleen or liver or both are not included.

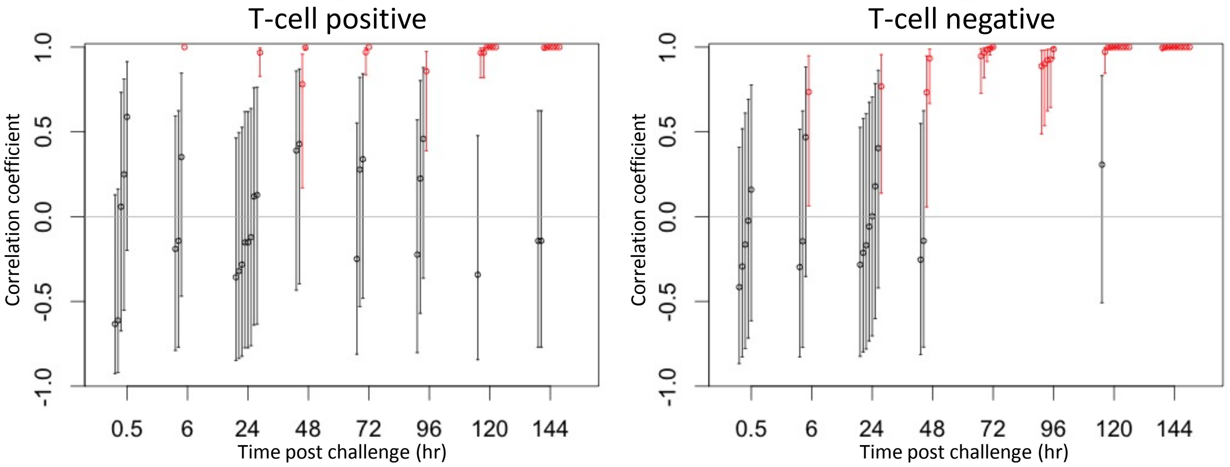

Supplement: Figure S8 — Hepatic and splenic WITS population correlation coefficients for LV-immunised, T-cell positive and T-cell negative groups. (PDF) [file ppat.1004359.s008.pdf]
