# Supplementary material for: The Effects of Vaccination and Immunity on Bacterial Infection Dynamics In Vivo
Source: PLoS Pathog. 2014 Sep 18;10(9):e1004359. doi: 10.1371/journal.ppat.1004359 (PMC4169467; doi:10.1371/journal.ppat.1004359)
Supplement: Table S1 — Experimental group sizes. (PDF) [file ppat.1004359.s009.pdf]

Table S1. Experimental group sizes.

Experimental groups	Group size at timepoint p.i. (hr)							
	0.5	6	24	48	72	96	120	144
Naive vs.	10	10	10	10	10	na	na	na
LV	9	9	9	9	9	na	na	na
Naive vs.	10	10	9	9	10	na	na	na
KV	10	10	10	10	10	na	na	na
LV T+ vs.	5	5	10	5	5	5	10	10
LV T-	5	5	10	5	5	5	10	10
