# Supplementary material for: The Effects of Vaccination and Immunity on Bacterial Infection Dynamics In Vivo
Source: PLoS Pathog. 2014 Sep 18;10(9):e1004359. doi: 10.1371/journal.ppat.1004359 (PMC4169467; doi:10.1371/journal.ppat.1004359)
Supplement: Table S3 — Primers used for qPCR. (PDF) [file ppat.1004359.s011.pdf]

Table S3: Primers used for qPCR: CC801 was used in combination with each of the others to amplify each specific tag.

Primer	Tag	Sequence (5’-3’)
CC793	1	ACGACACCACTCCACACCTA
CC794	2	ACCCGCAATACCAACAACTC
CC795	11	ATCCCACACACTCGATCTCA
CC796	13	GCTAAAGACACCCCTCACTCA
CC797	17	TCACCAGCCCACCCCCTCA
CC798	19	GCACTATCCAGCCCCATAAC
CC799	20	ACCTAACTATACCGCCATCC
CC800	21	ACAACCACCGATCACTCTCC
CC801	common	CACGGAAAACATCGTGAGTC
