# Supplementary material for: The Effects of Vaccination and Immunity on Bacterial Infection Dynamics In Vivo
Source: PLoS Pathog. 2014 Sep 18;10(9):e1004359. doi: 10.1371/journal.ppat.1004359 (PMC4169467; doi:10.1371/journal.ppat.1004359)
Supplement: Text S1 — Supplementary methods and results—stochastic model and inference. (PDF) [file ppat.1004359.s012.pdf]

The effects of vaccination and immunity on bacterial infection dynamics *in vivo*

Chris Coward, Olivier Restif, Richard Dybowski, Andrew J. Grant, Duncan J. Maskell, Pietro Mastroeni

Appendix: Supplementary methods and results – Stochastic model and inference.

1. Branching process

1.a. Model definition

The model tracks the number of copies of a single WITS in three locations simultaneously: blood (n_B), liver (n_L) and spleen (n_S). There are 6 transitions and 6 associated parameters:

Transition	Rate	Effect
1. Transfer from B to L	$c_L n_B$	$n_B \leftarrow n_B - 1$
		$n_L \leftarrow n_L + 1$
2. Transfer from B to S	$c_S n_B$	$n_B \leftarrow n_B - 1$
		$n_S \leftarrow n_S + 1$
3. Replication in L	$r_L n_L$	$n_L \leftarrow n_L + 1$
4. Replication in S	$r_S n_S$	$n_S \leftarrow n_S + 1$
5. Killing in L	$k_L n_L$	$n_L \leftarrow n_L - 1$
6. Killing in S	$k_S n_S$	$n_S \leftarrow n_S - 1$

As explained in the main body of the paper, we only used the model for the first 24 h of infection, when it is apparent from the data that there is no movement of bacteria out of the organs.

The dynamics of the model can be described by its master differential equation (Kolmogorov's forward equation):

$$\begin{aligned}\dot{P}(n_B, n_L, n_S) = \; & c_L P(n_B + 1, n_L - 1, n_S)(n_B + 1) + c_S P(n_B + 1, n_L, n_S - 1)(n_B + 1) \\ & + r_L P(n_B, n_L - 1, n_S)(n_L - 1) + r_S P(n_B, n_L, n_S - 1)(n_S - 1) \\ & + k_L P(n_B, n_L + 1, n_S)(n_L + 1) + k_S P(n_B, n_L, n_S + 1)(n_S + 1) \\ & - \left[(c_L + c_S)n_B + (r_L + k_L)n_L + (r_S + k_S)n_S\right] P(n_B, n_L, n_S)\end{aligned}$$

From this master equation it is possible to derive a closed system of 9 ordinary differential equations for the first two moments of the probability distribution, which we write in terms of the marginal expected values $E(N_B)$, $E(N_L)$, $E(N_S)$, variances $V(N_B)$, $V(N_L)$, $V(N_S)$ and covariances $V(N_B, N_L)$, $V(N_B, N_S)$, $V(N_L, N_S)$.

- Equation for the expectations in matrix form: $\dot{\mathbf{N}} = \mathbf{A} * \mathbf{N}$, with

$$\mathbf{N} = \begin{pmatrix} \mathbf{E}N_B \\ \mathbf{E}N_L \\ \mathbf{E}N_S \end{pmatrix}, \mathbf{A} = \begin{pmatrix} -(c_L + c_S) & 0 & 0 \\ cL & r_L - k_L & 0 \\ cS & 0 & r_S - k_S \end{pmatrix}$$

which can be solved as $\mathbf{N}(t) = \exp(t\mathbf{A}) * \mathbf{N}(0)$.

- Equation for the variances: Let $\mathbf{M} = {}^T\left[\mathbf{V}(N_B), \mathbf{V}(N_L), \mathbf{V}(N_S), \mathbf{V}(N_B, N_L), \mathbf{V}(N_B, N_S), \mathbf{V}(N_L, N_S)\right]$. Then: $\dot{\mathbf{M}}(t) = \mathbf{B} * \mathbf{N}(t) + \mathbf{C} * \mathbf{M}(t)$

$$\mathbf{B} = \begin{pmatrix} c_L + c_S & 0 & 0 \\ c_L & r_L + k_L & 0 \\ c_S & 0 & r_S + k_S \\ -c_L & 0 & 0 \\ -c_S & 0 & 0 \\ 0 & 0 & 0 \end{pmatrix}$$

$$\mathbf{C} = \begin{pmatrix} -2c_L - 2c_S & 0 & 0 & 0 & 0 & 0 \\ 0 & 2r_L - 2k_L & 0 & 2c_L & 0 & 0 \\ 0 & 0 & 2r_S - 2k_S & 0 & 2c_S & 0 \\ c_L & 0 & 0 & -c_L - c_S + r_L - k_L & 0 & 0 \\ c_S & 0 & 0 & 0 & -c_L - c_S + r_S - k_S & 0 \\ 0 & 0 & 0 & c_L & c_S & -k_L - k_S + r_L + r_S \end{pmatrix}$$

We can solve the equation: $\dot{\mathbf{M}}(t) - \mathbf{C} * \mathbf{M}(t) = \mathbf{B} * \exp(t\,\mathbf{A}) * \mathbf{N}(0)$ using Duhamel's formula.

The general solution is $\mathbf{M}(t) = \exp(t\,\mathbf{C}) * \mathbf{M}(0) + \exp(t\mathbf{C}) * \left[\int_0^t \exp(-s\mathbf{C}) * B * \exp(s\mathbf{A}) ds\right] * \mathbf{N}(0)$.

1.b. Initial conditions

All experiments were started by injecting small numbers of bacteria into the blood of mice. The exact inoculum sizes received by each mouse are unknown, but in each experiment a few inoculum doses, sampled from an even mix of the eight WITS, were plated to estimate the number of bacteria. Across experiments, inoculum sizes ranged from 25 to 50 cfu per WITS. We considered three alternative models with different initial conditions, which were all fitted to the data and compared using Akaike's Information Criterion:

- **Blood-fixed**: The initial number of copies of a single WITS in the blood followed a Poisson distribution truncated to its 99% upper quantile and centred on the mean inoculum size for that experiment.
- **Blood-inferred**: Here we assumed that the mean inoculum size was unknown and it was added to the list of parameters to be estimated. A truncated Poisson distribution was used with unknown mean i_B, raising the number of unknown parameters to 7.
- **Organs-inferred** For those experiments where no bacteria were recovered from the blood after 30 minutes, there was no upper bound for the migration parameters c_L and c_S. Thus we considered a model where unknown numbers of bacteria were transferred directly to the liver and the spleen at $t = 0$, with no bacteria remaining in the blood (hence the values of c_L and c_S became irrelevant and we set them to zero). Both initial numbers in the liver and spleen were assumed to follow Poisson distributions with unknown means x_L and x_S. Hence this model had 6 unknown parameters.

1.c. Numerical solution

Even though the master equation is a system of linear ordinary differential equations, it can involve several million equations in order to track up to a few hundred bacteria in each compartment. We found that the most efficient method to numerically solve the system was using a Runge-Kutta algorithm (as implemented in Numerical Recipes [S1]) where the number of equations was controlled dynamically using a meta-dynamical algorithm [S2].

To explain this process, let us consider the infinite three-dimensional array of model variables $P(n_B, n_L, n_S)$, for $n_B \geq 0, n_L \geq 0, n_S \geq 0$ At any given time t, if a given variable $P(n_B, n_L, n_S) = 0$, its value can only increase if any of the "neighbouring" variables listed in the master equation above is positive. Consequently, as the Runge Kutta unfolds in small time steps, the array slowly fills up with non-zero values. Instead of starting the algorithm with an arbitrary large array full of zeroes and updating every element at every step, it was more efficient to keep track of which variables were non-zero and include their direct neighbours at every time step. New variables were only added if their values exceeded an arbitrary threshold $\epsilon \ll 1$.

Given a threshold ϵ, at the current time t:

1. any variable $P(n_B, n_L, n_S) > \epsilon$ is deemed *active*
2. any variable (unless active itself) neighbouring an active variable is deemed *passive*
3. all other variables are deemed *inactive* and their value set to 0
4. calculate the derivatives of active and passive variables (Runge-Kutta) and update their values
5. any passive variable whose value is now > ϵ is set to active
6. update the list of passive variables using the new list of active variables
7. go back to 4

We tested the accuracy of the method across a range of ϵ values and arbitrary sets of parameter values, by comparing the means and variances of the numerical solution $P_t^\epsilon(n_B, n_L, n_S)$ with the values expected from the master equation (section 1.a above). Based on the results shown in Figure A1, we used $\epsilon = 10^{-9}$ for final results and higher values for exploratory analyses.

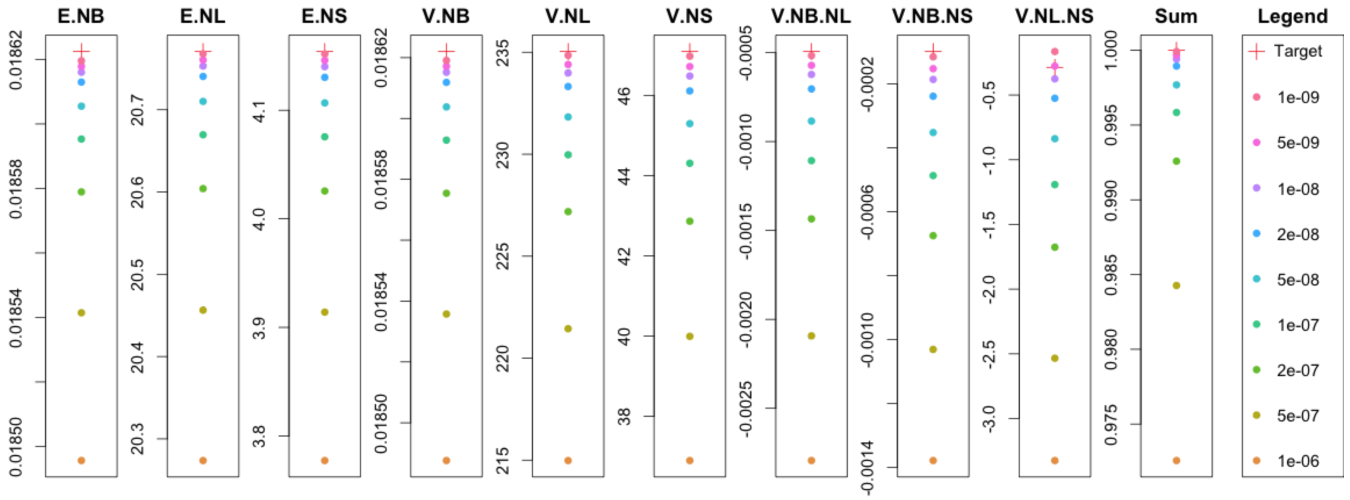

Figure A1. The first 9 panels show the 9 statistics, with the red cross indicating the target value calculated by solving the moments equations, and the coloured dots showing the values obtained from the metadynamical algorithm with decreasing values of ϵ from 10^{-6} to 10^{-9}. The last panel ('Sum') shows the sum of all the variables $P_t^{\epsilon}(n_B, n_L, n_S)$, which should be as close to 1 as possible. Parameter values used: $c_L = 1, c_S = 0.2, e_L = 0, e_S = 0, k_L = 1, k_S = 1, r_L = 1, r_S = 1$; initial conditions: bacteria in the blood only following a Poisson distribution with mean 25 capped at the 99.5% top quantile.

2. Inference method

2.a. Likelihood function

Given parameter values Θ and initial conditions $\hat{P}_0$, the model above gives the probability distribution of system state $N_t = (n_B, n_L, n_S)$ of the system. The set of observations at time t in mouse m is:

$$\Omega_{t,m} = \left(x^B, x^L, x^S, p_1^B, \ldots, p_7^B, p_1^L, \ldots, p_7^L, p_1^S, \ldots, p_7^S; \varphi^B, \varphi^L, \varphi^S\right)$$
$$= \left(\omega_1^B, \ldots, \omega_8^B, \omega_1^L, \ldots, \omega_8^L, \omega_1^S, \ldots, \omega_8^S; \varphi^B, \varphi^L, \varphi^S\right)$$

where x represents the cfu counted from a fraction φ of the organ, p_k is the proportion of WITS k in the whole sample after plate growth and qPCR, and $\omega_k^i \equiv p_k^i x^i$, with $p_8 \equiv 1 - \sum_{k=1}^{7} p_k$.

We assume that all WITS in a mouse are identical and independent of each other. In addition, since the observation process is carried out on individual organs separately, we assume that, given (n_B, n_B, n_S), the observations $\omega_b, \omega_L, \omega_S$ are independent of each other.

The likelihood of Θ is equal to:

$$L_{t,m}(\Theta) = f(\Omega_{t,m}|\Theta)$$
$$= \prod_{k=1}^{8} f(\omega_k^B, \omega_k^L, \omega_k^S|\Theta)$$
$$= \prod_{k=1}^{8}\left[\sum_{n_B,n_L,n_S} f(\omega_k^B, \omega_k^L, \omega_k^S|n_B, n_L, n_S)P(n_B, n_L, n_S|\Theta)\right]$$
$$= \prod_{k=1}^{8}\left[\sum_{n_B,n_L,n_S} f(\omega_k^B|n_B)f(\omega_k^L|n_L)f(\omega_k^S|n_S)P(n_B, n_L, n_S|\Theta)\right]$$

The last factor (P) inside the sum is given by the dynamic model, while the first factors (f) describe the experimental observation process.

2.b Observation process

In order to estimate the overall level of variation in the experimental process, we ran a calibration experiment as follows: we plated colonies from each WITS on separate plates, incubated for the same amount of time. Known numbers (ranging from 1 to 100) of colonies from all 8 WITS were mixed and processed by qPCR following the same protocol as described in the Methods section. Thus, the colonies on each plate were treated in exactly the same way as the bacteria extracted from mouse organs in the main experiment. The proportions of the 8 WITS estimated by qPCR were multiplied by the total number of colonies that had been combined together. In line with the notations used above, this product is noted ω_i^k for WITS i on plate k, and compared to the actual number of colonies n_i^k of WITS i that were present on plate k (Fig. A1).

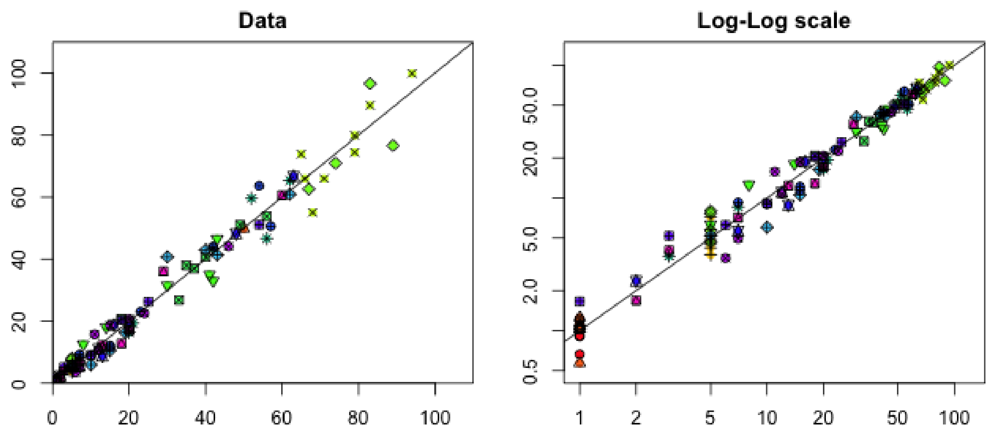

Figure A2. Data from 14 plates: for each WITS from each plate, the actual number of colonies n is plotted on the horizontal axis (cfu) against the number of copies ω inferred from qPCR on the vertical axis. Left: linear scales, right: log scales.

We then fitted by maximum likelihood, and compared by AIC, six alternative models for the distribution $f(\omega|n)$, where n is the number of colonies of each WITS on a plate and ω is the "number of copies" inferred from qPCR (proportions * total number of cfu).

1. $\omega \sim \mathcal{N}(n, \alpha)$
2. $\omega \sim \mathcal{N}(n, \alpha n^{\beta})$
3. $\omega \sim \mathcal{N}(n, \alpha(1 - e^{-\beta n}))$
4. $log(\omega) \sim \mathcal{N}(log(n), \alpha)$
5. $log(\omega) \sim \mathcal{N}(log(n), \alpha n^{\beta})$
6. $log(\omega) \sim \mathcal{N}(log(n), \alpha e^{-\beta n})$

In each model, α and β were estimated by maximum likelihood.

Results from the AIC comparison show that the last model (6) is best. The table below shows the difference in AIC between each model and model (6). Larger values indicate decreasing support.

```
  Model     AIC
1     1 1981.81
2     2  563.11
3     3  555.28
4     4   25.23
5     5    6.74
6     6    0.00
```

Best model for $f(\omega|n)$: (6) log-normal distribution with log-standard-deviation = 0.2674 exp(-0.0148 n). We then used this function to calculate the likelihood of the stochastic model as described in the previous section.

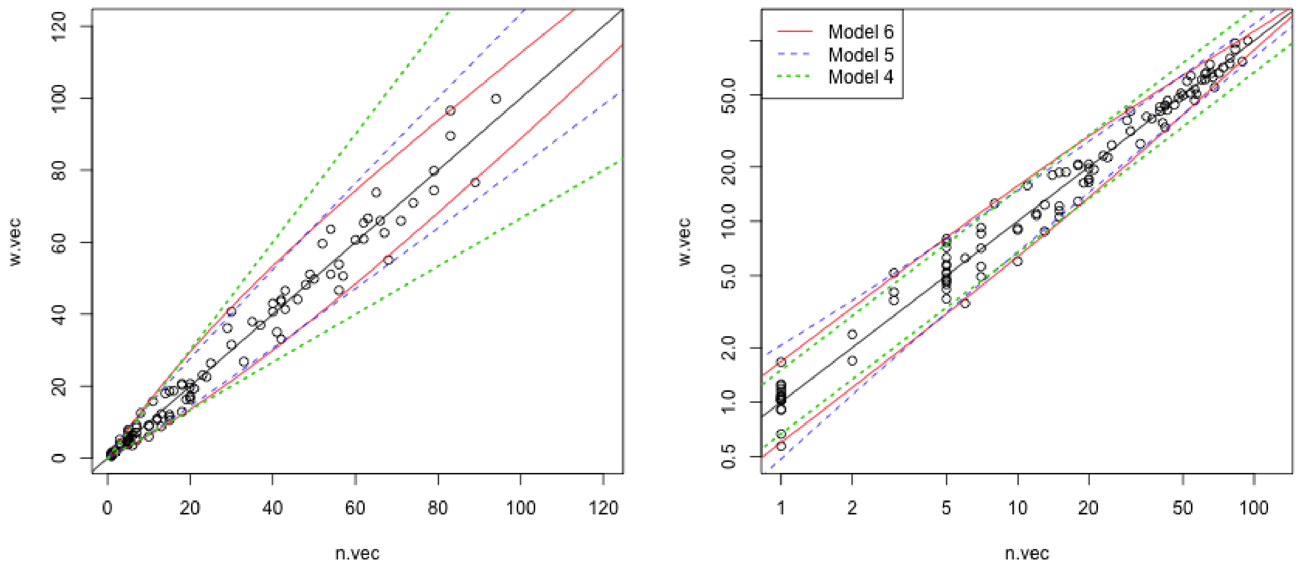

Figure A3: Same data as in Fig. A2, showing 95% confidence intervals for ω as afunction of n, from fitted models (4), (5) and (6). Left: linear scales, right: log scales.

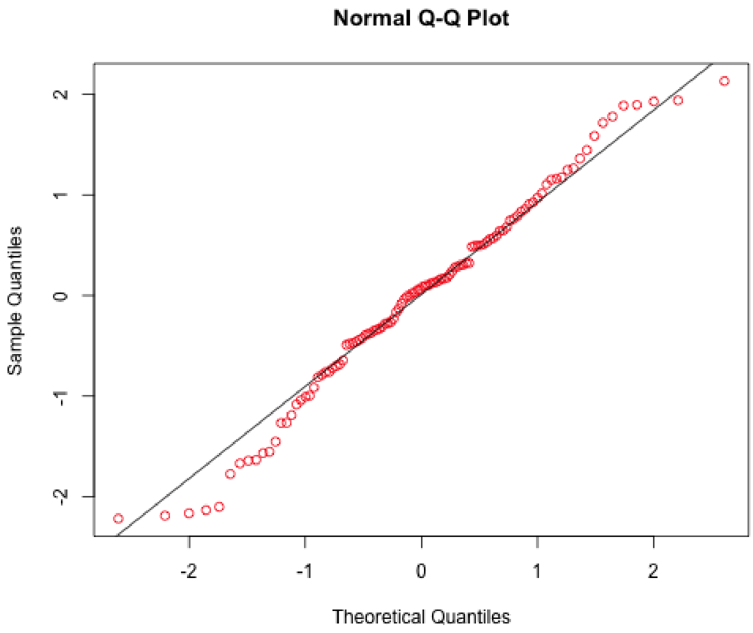

Figure A4: Goodness of fit of model (6) assessed using a normal Q-Q plot.

2.c. Parameter estimation and confidence intervals using maximum likelihood

The parameters of the stochastic model for bacterial dynamics were estimated by maximum likelihood, using the Powell package in R [S3], which implements unconstrained optimization by quadratic approximation [S4].

We repeatedly performed maximum likelihood computation on each dataset with decreasing values of ϵ, starting from 10^{-7}, until successive values of the log-likelihood maximum changed by less than 0.1 unit.

Elliptic confidence regions for parameter estimates were computed using a normal approximation of the likelihood function: the inverse of the Fisher Information $I(\hat{\Theta})$, defined as the Hessian matrix of $-logL(\hat{\Theta})$ at the MLE, provides the covariance matrix of $\hat{\Theta}$, from which 95% confidence intervals and multivariate confidence ellipsoids can be estimated.

3. Supplementary results

3.a. Parameter estimates from 0 to 6h

We assumed parameters remained constant for the first 6h, and fit model to data at both t=0.5h and t=6h.

Model comparison

Table of AICc for each experimental group:

	Experiment	Treatment	Model	AIC
1	Live vaccine	Naive	blood-fixed	2164
2	Live vaccine	Naive	blood-inferred	2150
3	Live vaccine	Naive	organs-inferred	NA
4	Live vaccine	Immunised	blood-fixed	1295
5	Live vaccine	Immunised	blood-inferred	1135
6	Live vaccine	Immunised	organs-inferred	1135
7	Killed vaccine	Naive	blood-fixed	2252
8	Killed vaccine	Naive	blood-inferred	2186
9	Killed vaccine	Naive	organs-inferred	NA
10	Killed vaccine	Immunised	blood-fixed	1493
11	Killed vaccine	Immunised	blood-inferred	1485
12	Killed vaccine	Immunised	organs-inferred	1484
13	T-cells	+	blood-fixed	NA
14	T-cells	+	blood-inferred	NA
15	T-cells	+	organs-inferred	1481
16	T-cells	-	blood-fixed	NA
17	T-cells	-	blood-inferred	NA
18	T-cells	-	organs-inferred	1499

Best models

Inoculum sizes:

	Experiment	Treatment	Inoculum	Predicted	Pred.min	Pred.max	Loss	Liver
1	Live vaccine	Naive	243	228	219	237	0.063	0.75
2	Live vaccine	Immunised	243	140	127	153	0.424	0.85
3	Killed vaccine	Naive	280	214	205	222	0.237	0.76
4	Killed vaccine	Immunised	280	261	247	275	0.069	0.92
5	T-cells	+	355	274	266	281	0.228	0.91
6	T-cells	-	355	274	273	274	0.229	0.88

Killing (expressed as fraction of bacteria killed per hour):

	Experiment	Treatment	Liver	Spleen
1	Live vaccine	Naive	0.56	0.27
2	Live vaccine	Immunised	0.54	0.45
3	Killed vaccine	Naive	0.57	0.35
4	Killed vaccine	Immunised	0.71	0.27
5	T-cells	+	0.74	0.65
6	T-cells	-	0.66	0.87

Killing (expressed as half-life):

	Experiment	Treatment	Liver	Spleen
1	Live vaccine	Naive	0.84	2.23
2	Live vaccine	Immunised	0.90	1.15
3	Killed vaccine	Naive	0.82	1.62
4	Killed vaccine	Immunised	0.57	2.21
5	T-cells	+	0.52	0.66
6	T-cells	-	0.64	0.34

Replication rate (expressed as relative increase per hour):

	Experiment	Treatment	Liver	Spleen
1	Live vaccine	Naive	0.71	0.31
2	Live vaccine	Immunised	0.66	0.47
3	Killed vaccine	Naive	0.74	0.41
4	Killed vaccine	Immunised	1.31	0.19
5	T-cells	+	1.62	1.77
6	T-cells	-	1.11	5.87

Replication (expressed as doubling time):

	Experiment	Treatment	Liver	Spleen
1	Live vaccine	Naive	1.30	2.57
2	Live vaccine	Immunised	1.37	1.80
3	Killed vaccine	Naive	1.25	2.00
4	Killed vaccine	Immunised	0.83	4.01
5	T-cells	+	0.72	0.68
6	T-cells	-	0.93	0.36

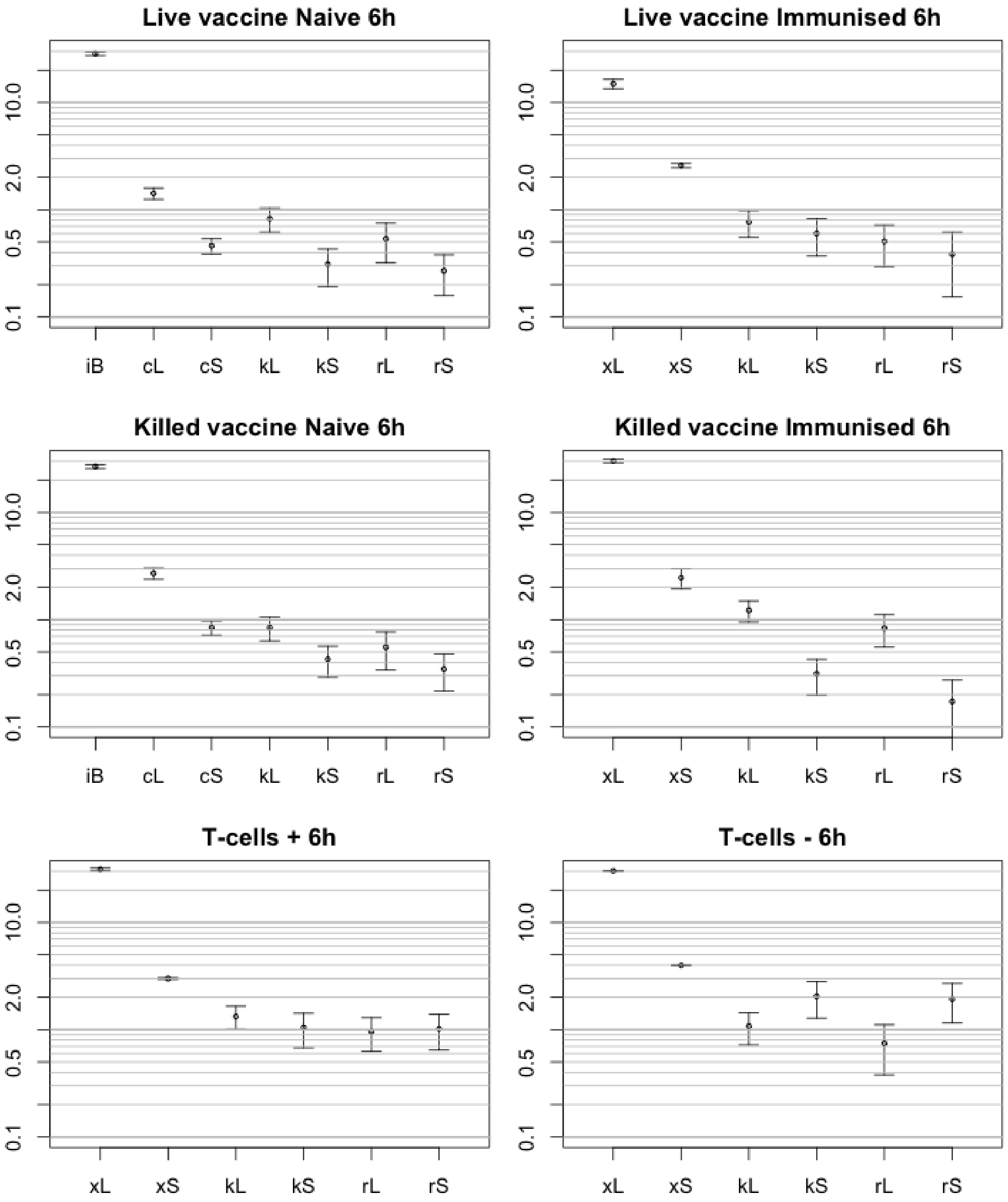

Figure A5. 95% Confidence intervals on parameter estimates based on normal approximation of the log-likelihood around its maximum.

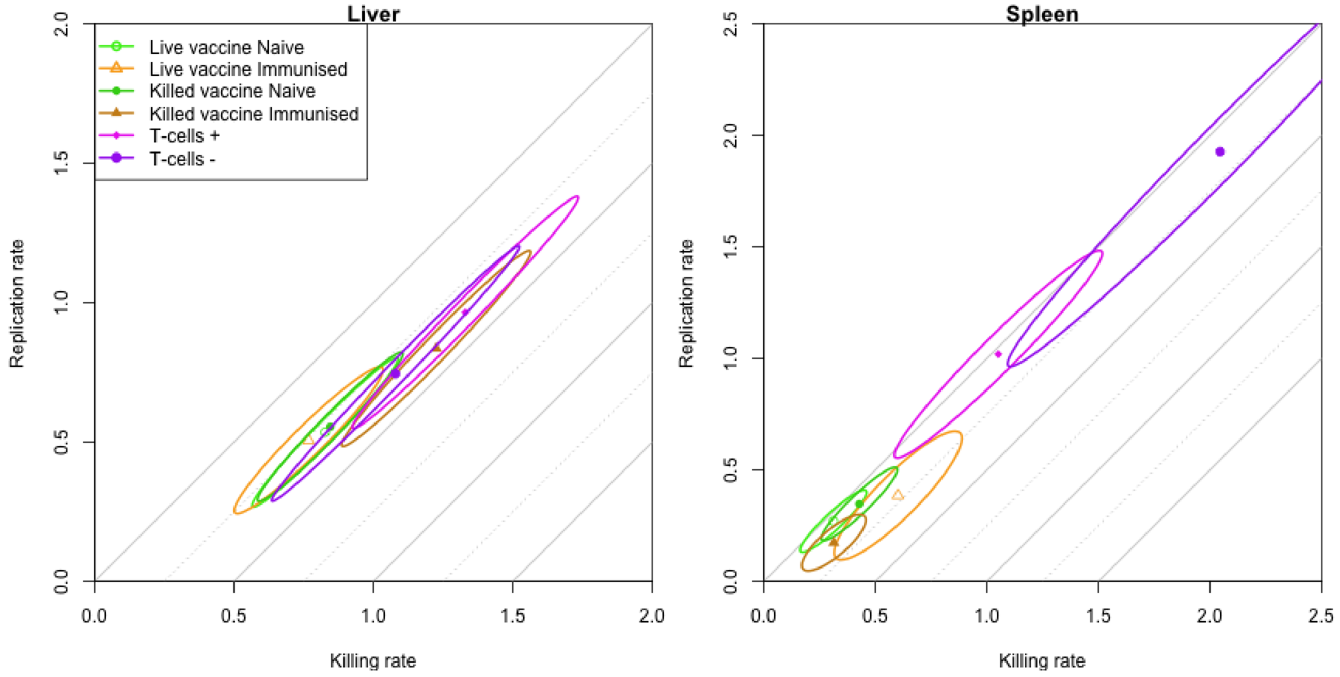

Figure A6. 95% bivariate confidence region for (r,k).

3.b. Goodness of fit from 0 to 6h

In order to assess the quality of the fitted model, we generated virtual mice by sampling randomly groups of 8 WITS from the output of the model, i.e. the distribution $P_t^{\epsilon}(n_B, n_L, n_S)$, to which we added a log-normal random noise representing the observation process. We generated 100 mice for each group, and produced three types of summary plots, shown below:

- boxplots of total CFU in the blood, liver and spleen at 0.5h and 6h post challenge;
- histograms of the numbers of WITS present in the blood, liver and spleen at each time point;
- paired scatter plot of the joint number of copies of each WITS found in the liver and spleen of each mouse (representing the raw data to which the model was fitted).

In all the figures below, the experimental data re shown in red and the simulated data are shown in blue.

Overall, the main discrepancy came from over-dispersed experimental data: the fitted model tends to predict less variability across mice or WITS than observed, even after taking into account the noise introduced by the observation process (culture and qPCR). This suggests additional variability between mice. We are planning to explore this in more detail using a Bayesian hierarchical framework.

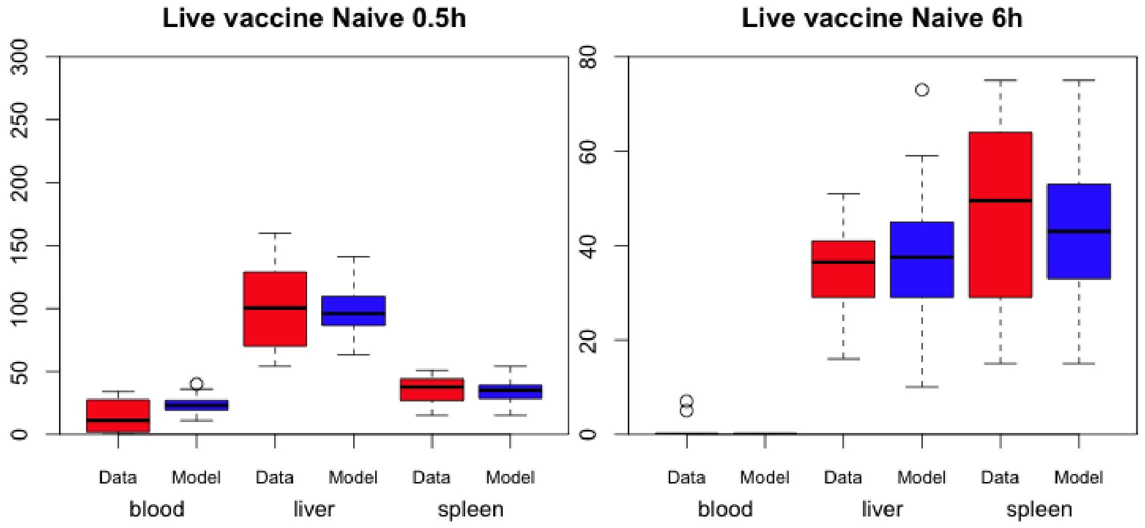

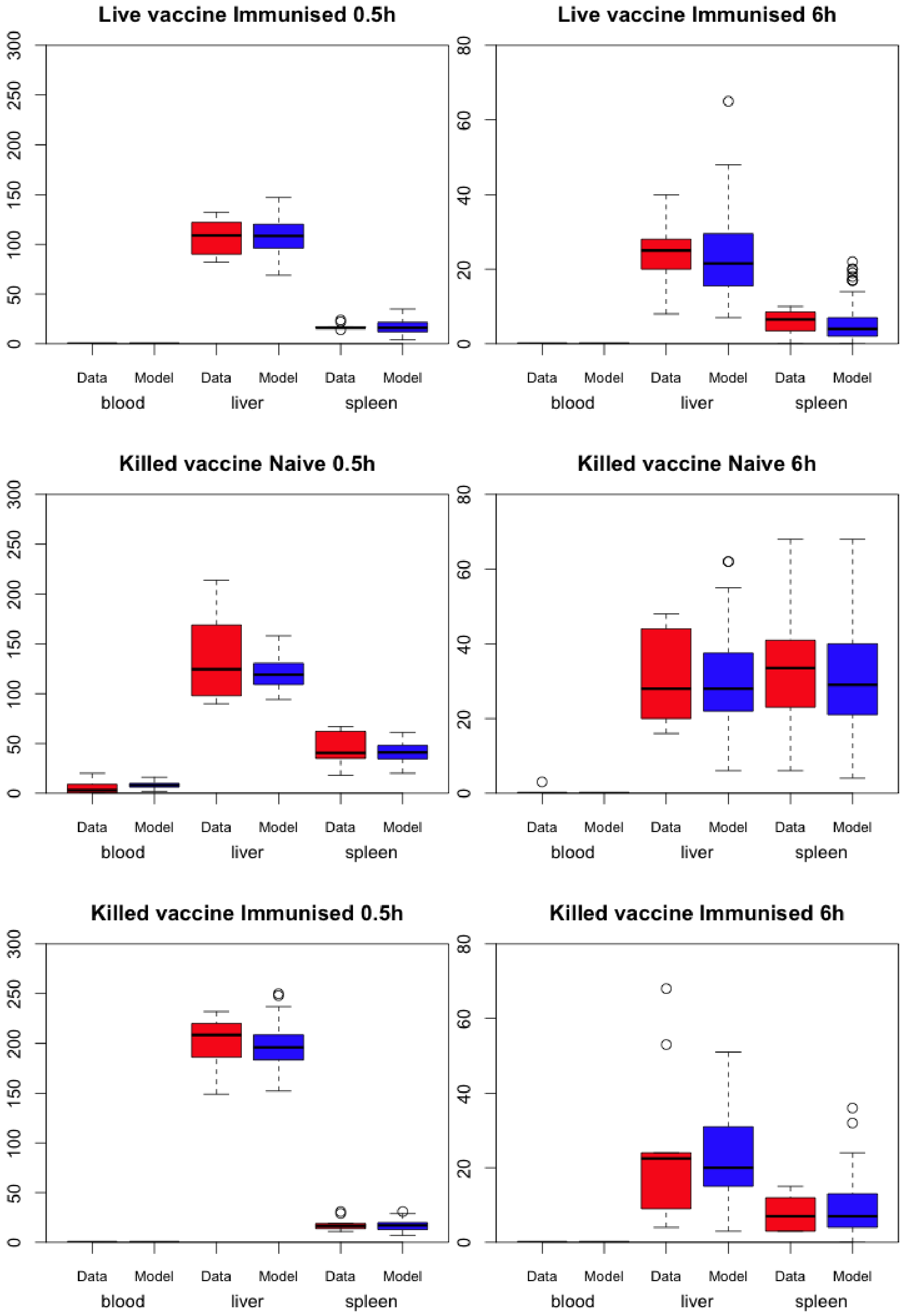
Live vaccine Immunised 0.5h
Live vaccine Immunised 6h
Killed vaccine Naive 0.5h
Killed vaccine Naive 6h
Killed vaccine Immunised 0.5h
Killed vaccine Immunised 6h
Data
Model
blood
liver
spleen
0
50
100
150
200
250
300
20
40
60
80

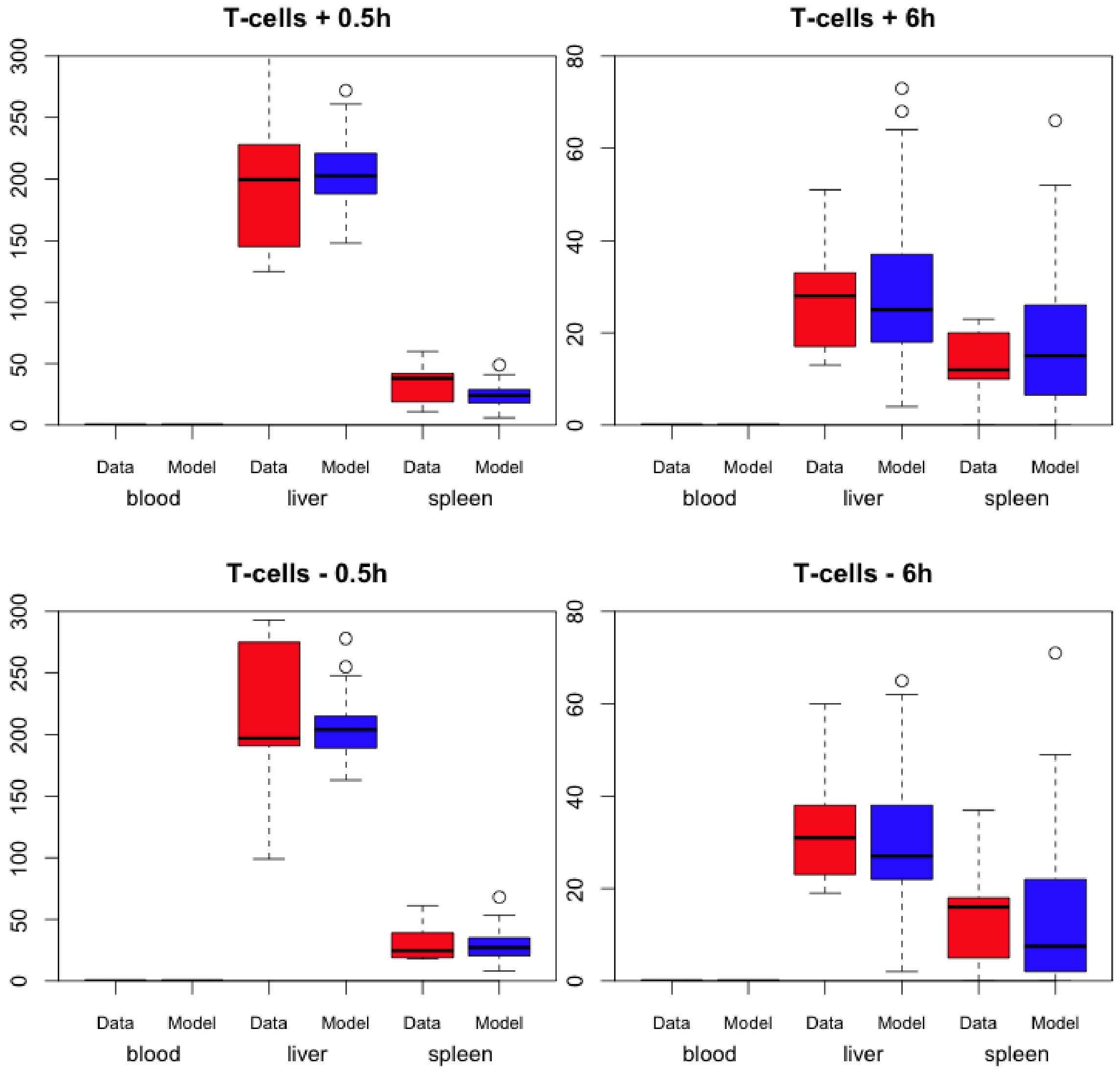

Figure A7. Observed and predicted total cfu at 0.5 and 6h in blood, liver and spleen (using experimental fractions of blood).

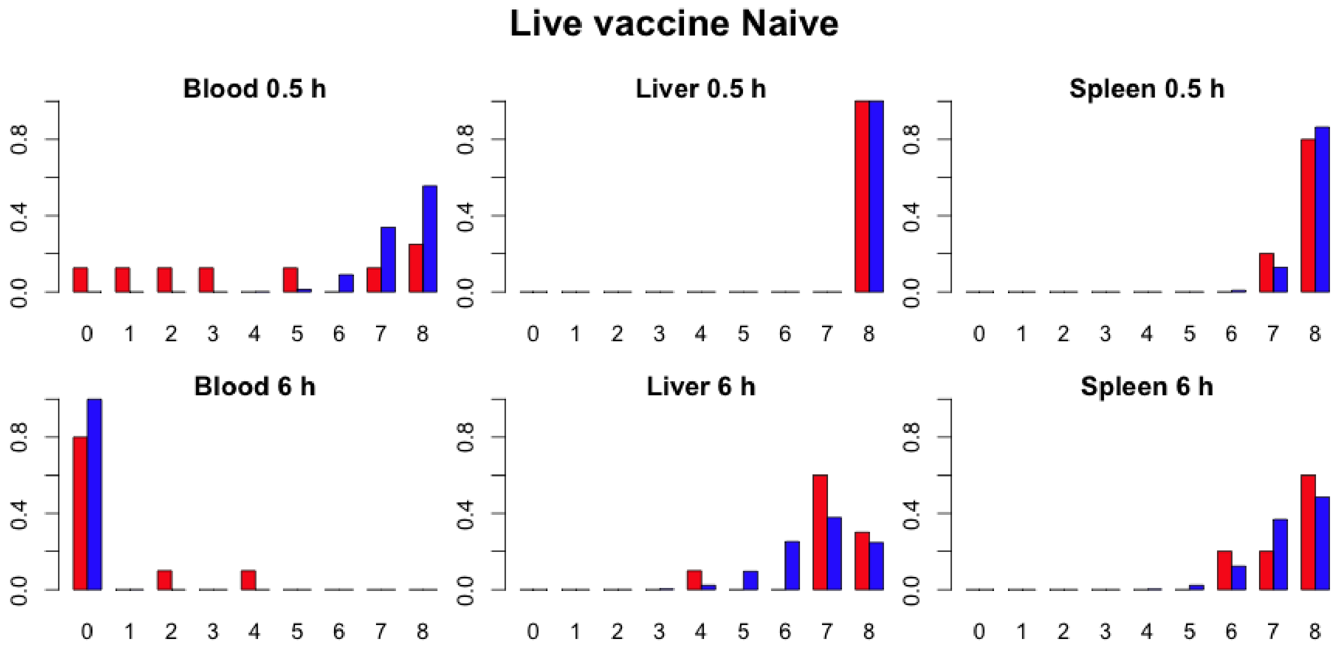

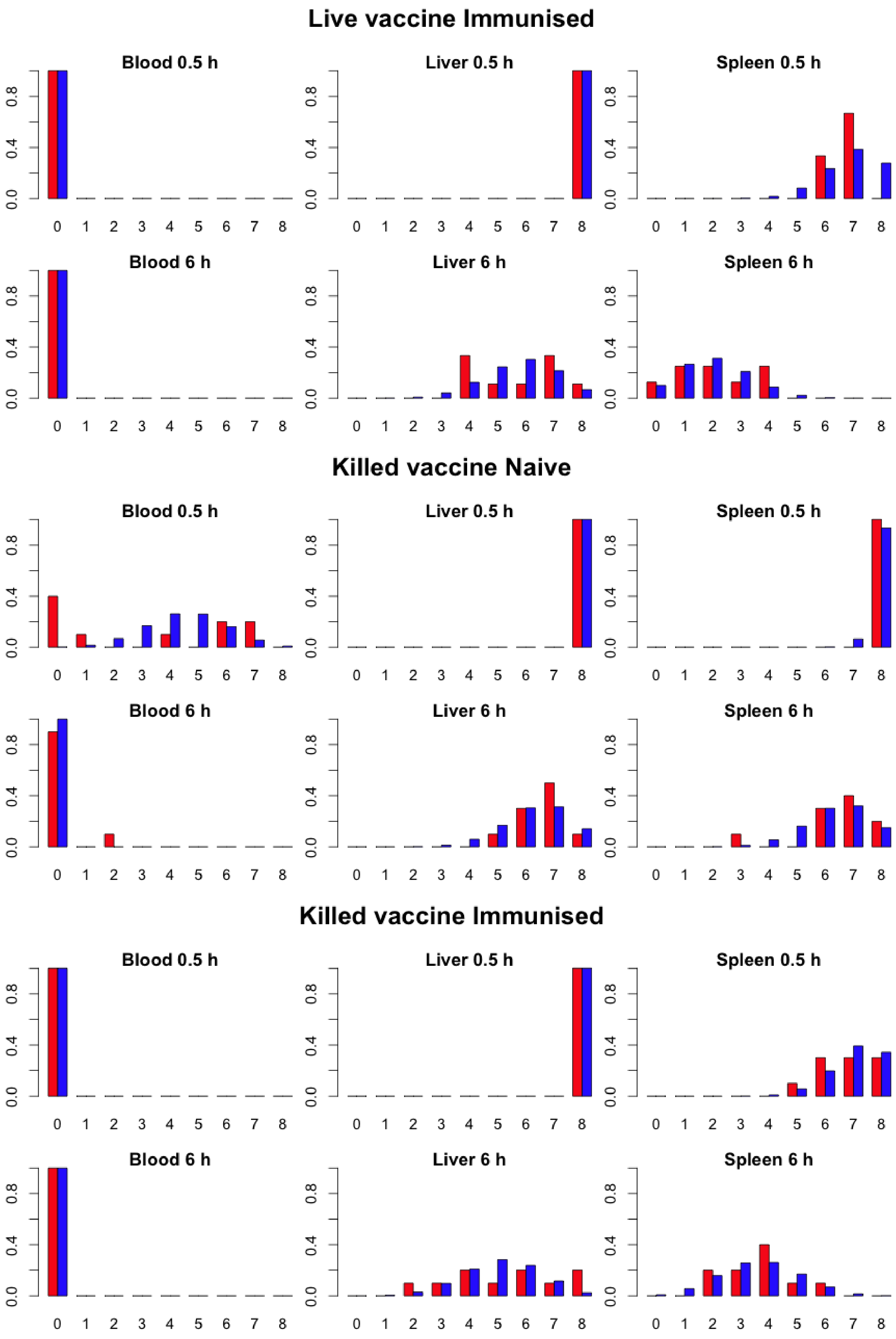
Live vaccine Immunised
Blood 0.5 h
Liver 0.5 h
Spleen 0.5 h
Blood 6 h
Liver 6 h
Spleen 6 h
Killed vaccine Naive
Blood 0.5 h
Liver 0.5 h
Spleen 0.5 h
Blood 6 h
Liver 6 h
Spleen 6 h
Killed vaccine Immunised
Blood 0.5 h
Liver 0.5 h
Spleen 0.5 h
Blood 6 h
Liver 6 h
Spleen 6 h

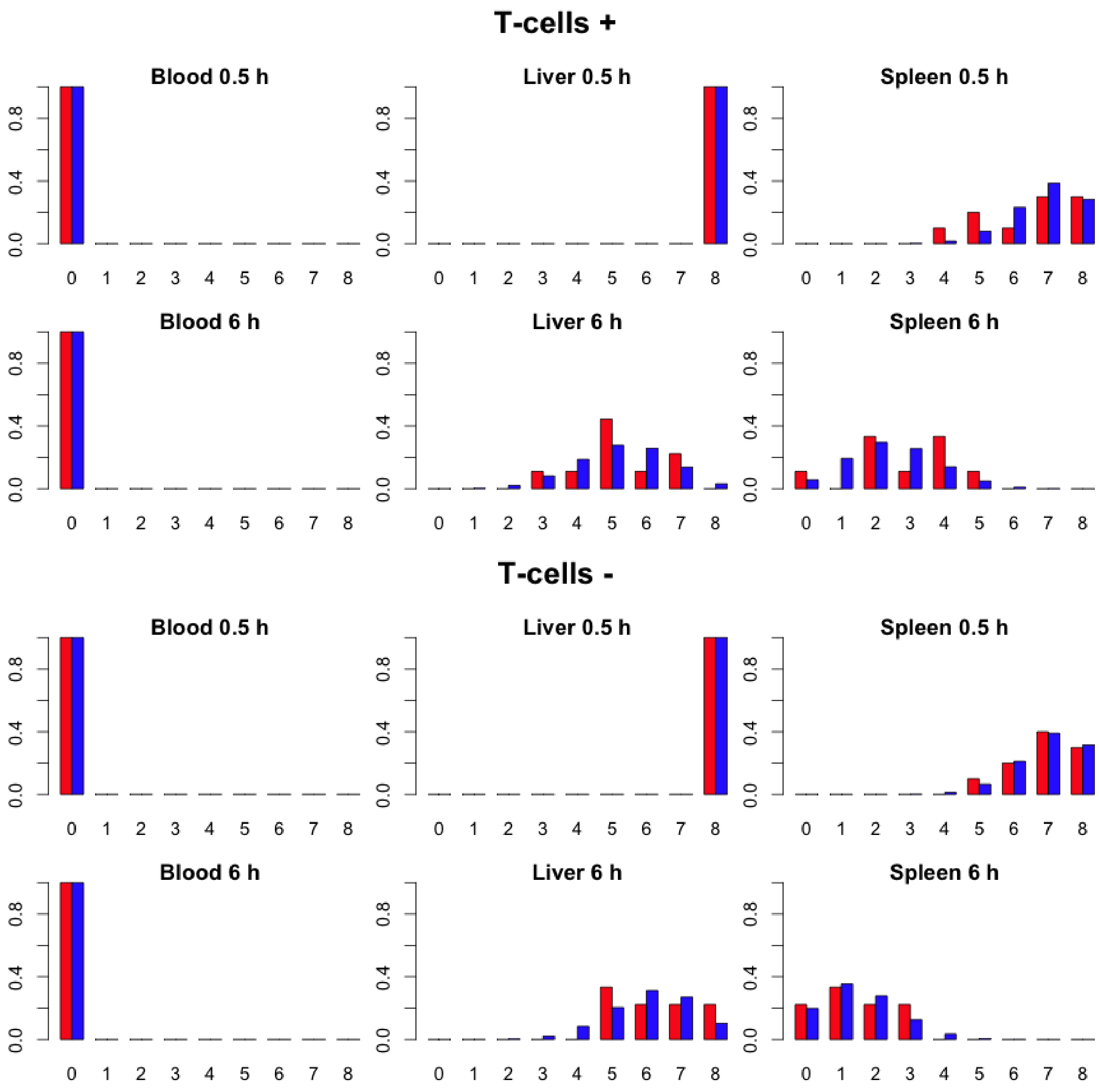

Figure A8. Histograms of the number of WITS present in each compartment (sampling from experimental fractions in the blood).

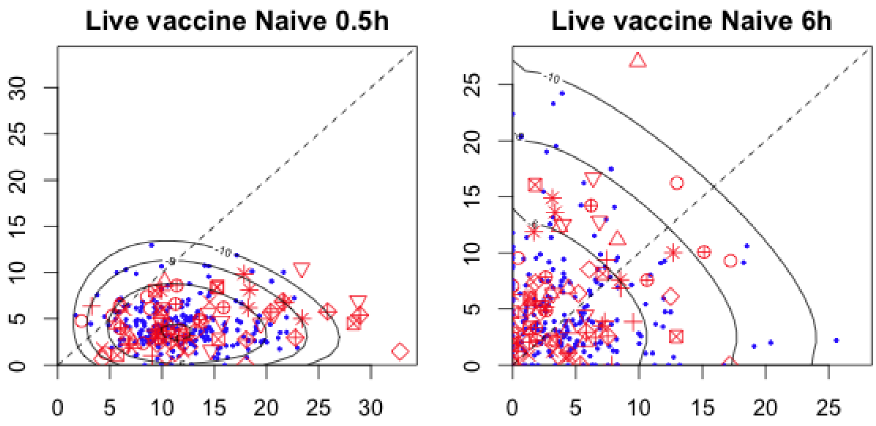

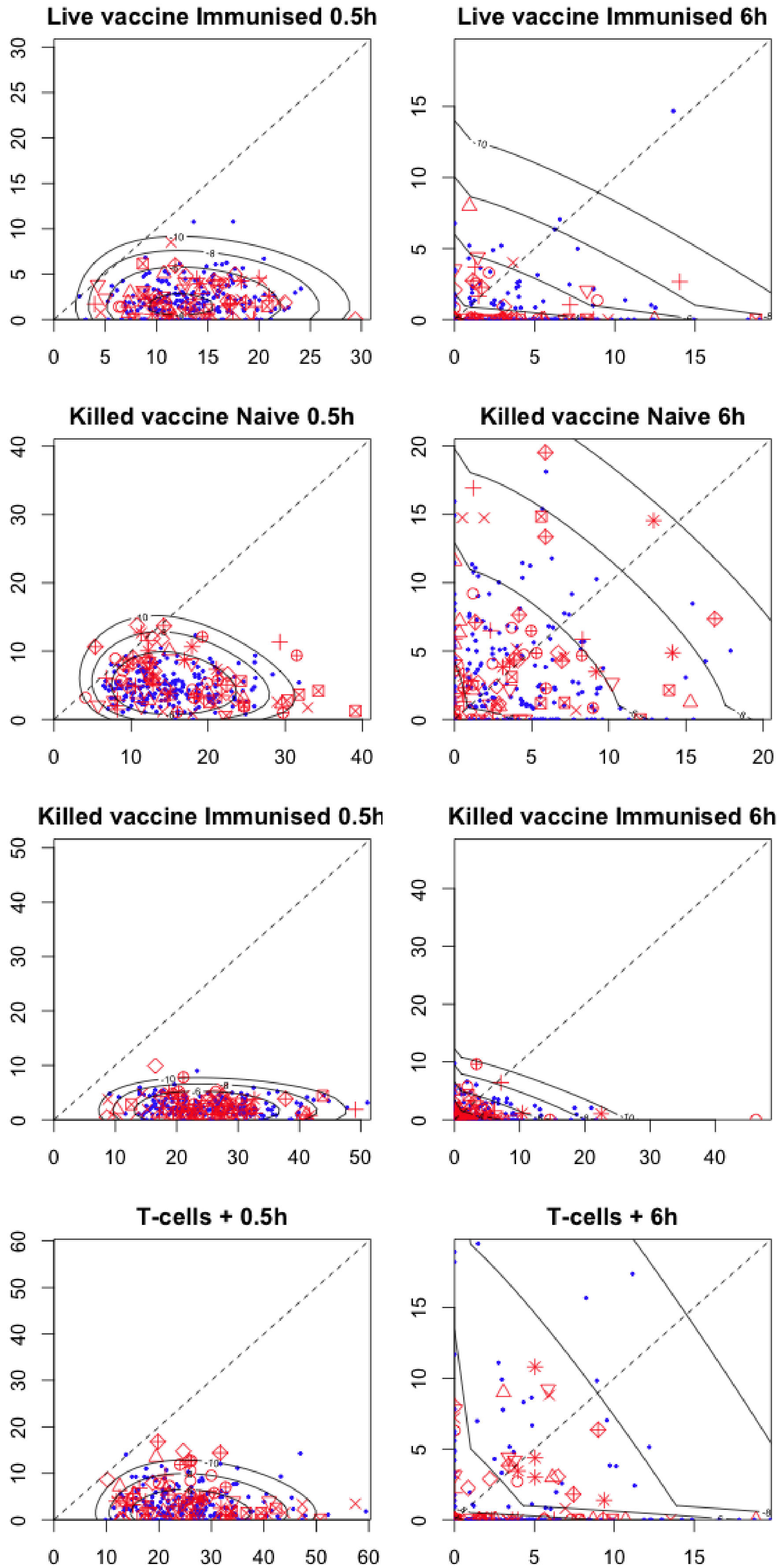
Live vaccine Immunised 0.5h
Live vaccine Immunised 6h
Killed vaccine Naive 0.5h
Killed vaccine Naive 6h
Killed vaccine Immunised 0.5h
Killed vaccine Immunised 6h
T-cells + 0.5h
T-cells + 6h

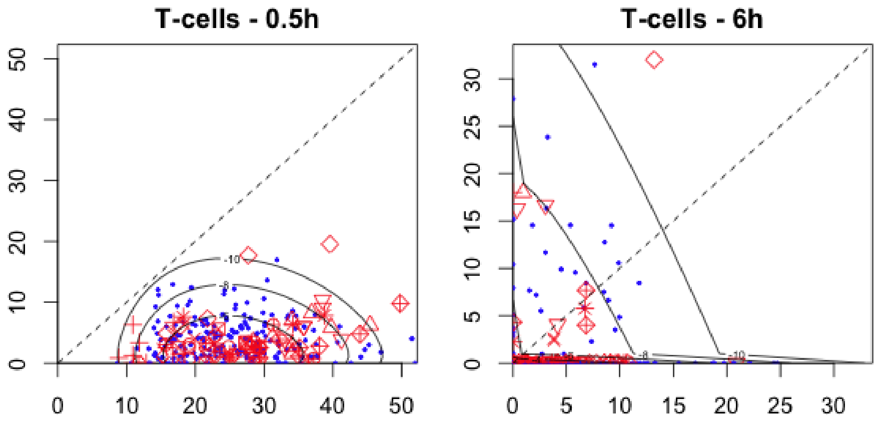

Figure A9. Absolute abundances of WITS in the liver (x-axis) and the spleen (y-axis). Each red symbol shows data from a single WITS in a single mouse from a given experimental group. Blue symbols show 100 simulated mice obtained by sampling the best fitted model at the maximum-likelihood estimate. Black lines are probability isoclines from the fitted model (labels show $log_{10} P$).

Correlation coefficients between liver and spleen predicted by the fitted models (see Figures S1 and S2 for comparison with experimental data):

	0.5h	6h
Live vaccine Naive	-6.958e-02	-0.0156853
Live vaccine Immunised	-1.918e-05	-0.0005317
Killed vaccine Naive	-9.491e-02	-0.0126418
Killed vaccine Immunised	-1.340e-05	-0.0001530
T-cells +	-9.981e-05	-0.0040111
T-cells -	-1.698e-04	-0.0047587

3.c. Parameter estimates from 6 to 24h.

Table of AIC for each experimental group. In order to test whether any significant bactericidal activity took place in the liver and the spleen after 6h post inoculation, we compared four alternative models:

- full model with 4 unknown parameters (k_L, k_S, r_L, r_S),
- no killing in the liver (3 parameters),
- no killing in the spleen (3 parameters),
- no killing in both organs (2 parameters).

	all	kL=0	kS=0	kL=kS=0
Live vaccine Naive	0.000e+00	3.937	35.618	39.48
Live vaccine Immunised	1.925e+00	20.733	0.000	18.81
Killed vaccine Naive	3.671e+02	0.000	3.202	362.95
Killed vaccine Immunised	0.000e+00	3.165	7.749	83.86
T-cells +	2.000e+09	1.820	NA	0.00
T-cells -	4.016e+00	1.896	NA	0.00

Killing rates (expressed as fraction of bacteria killed per hour):

	Experiment	Treatment	Liver	Spleen
1	Live vaccine	Naive	0.07	0.16
2	Live vaccine	Immunised	0.11	0.00
3	Killed vaccine	Naive	0.00	0.07
4	Killed vaccine	Immunised	0.07	0.12
5	T-cells	+	0.00	0.00
6	T-cells	-	0.00	0.00

Replication rate (a value of 1 means doubling every hour):

	Experiment	Treatment	Liver	Spleen
1	Live vaccine	Naive	0.230	0.306
2	Live vaccine	Immunised	0.178	0.091
3	Killed vaccine	Naive	0.107	0.213
4	Killed vaccine	Immunised	0.161	0.283
5	T-cells	+	0.024	0.027
6	T-cells	-	0.068	0.059

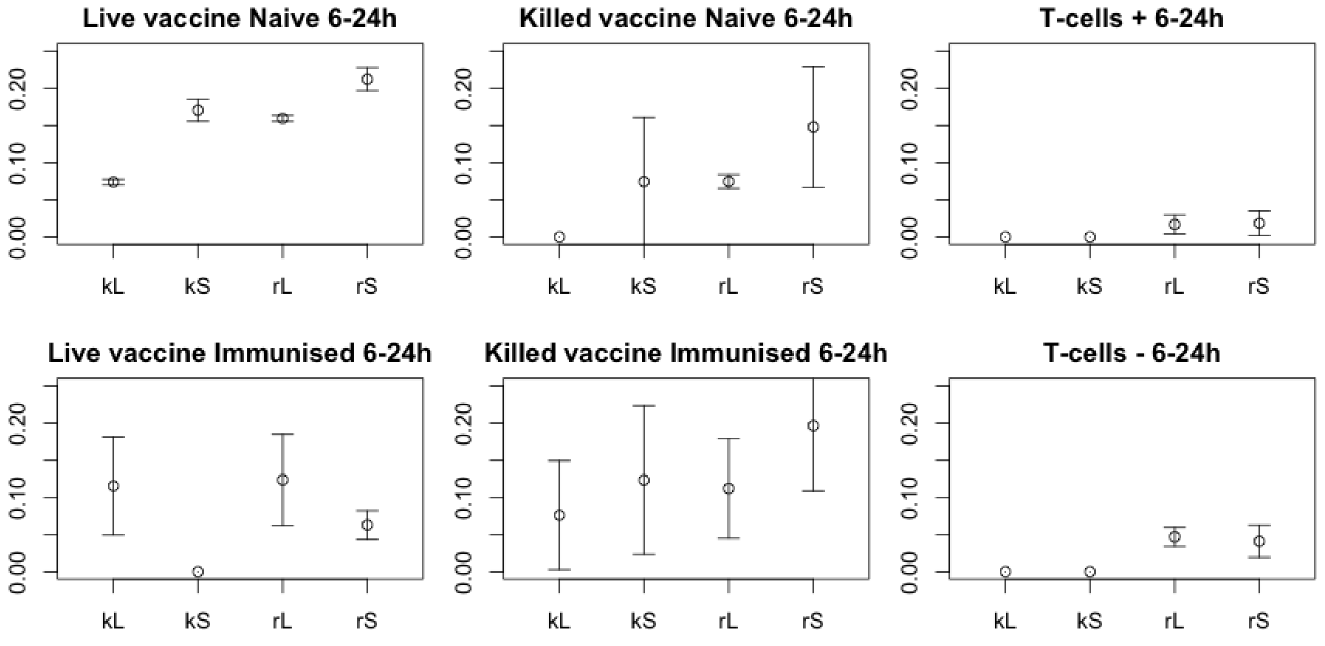

Figure A10. Parameter estimates with 95% confidence intervals.

3.d. Goodness of fit from 6h to 24h

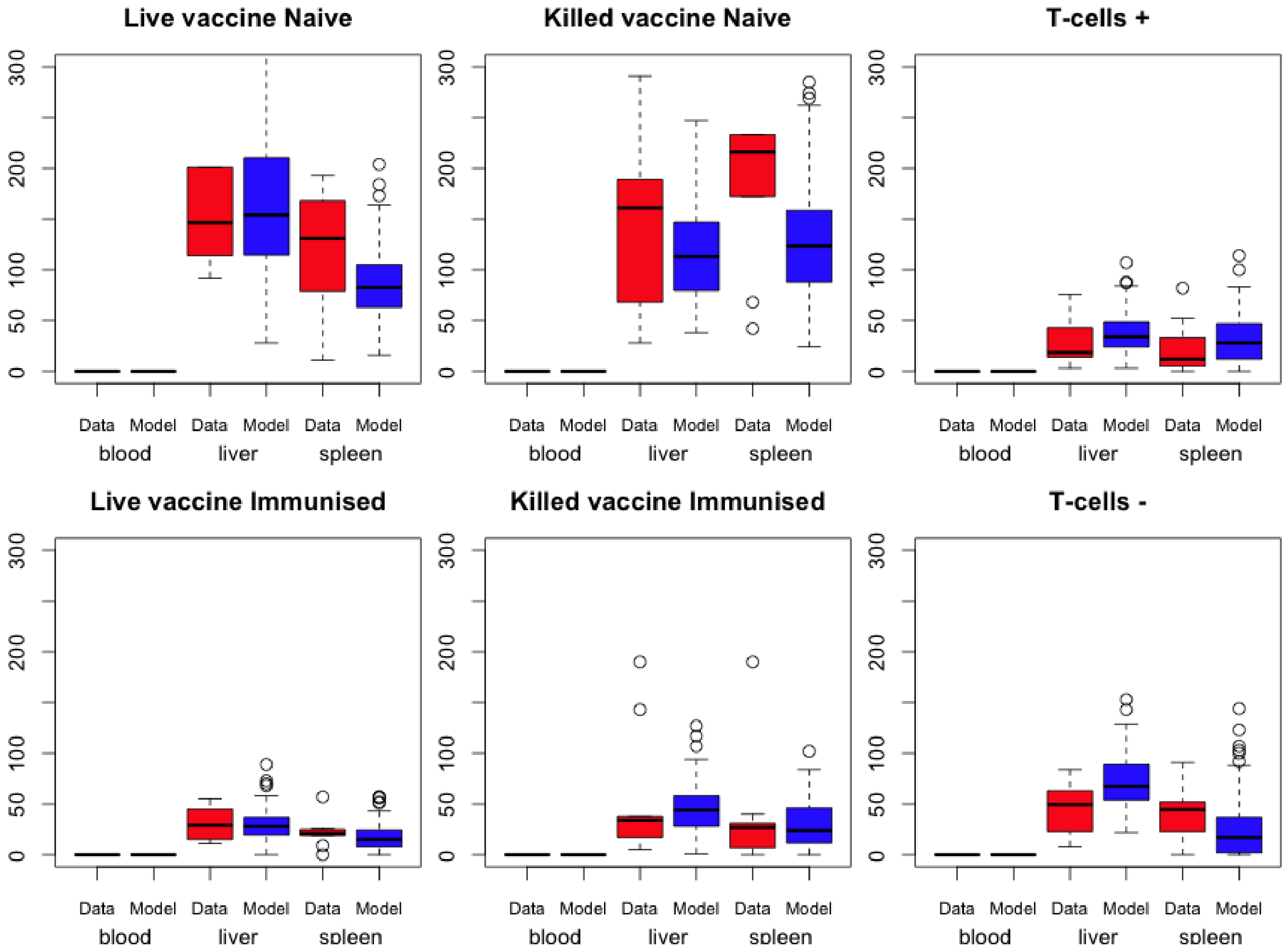

Figure A11. Observed and predicted total cfu at 24h in blood, liver and spleen.

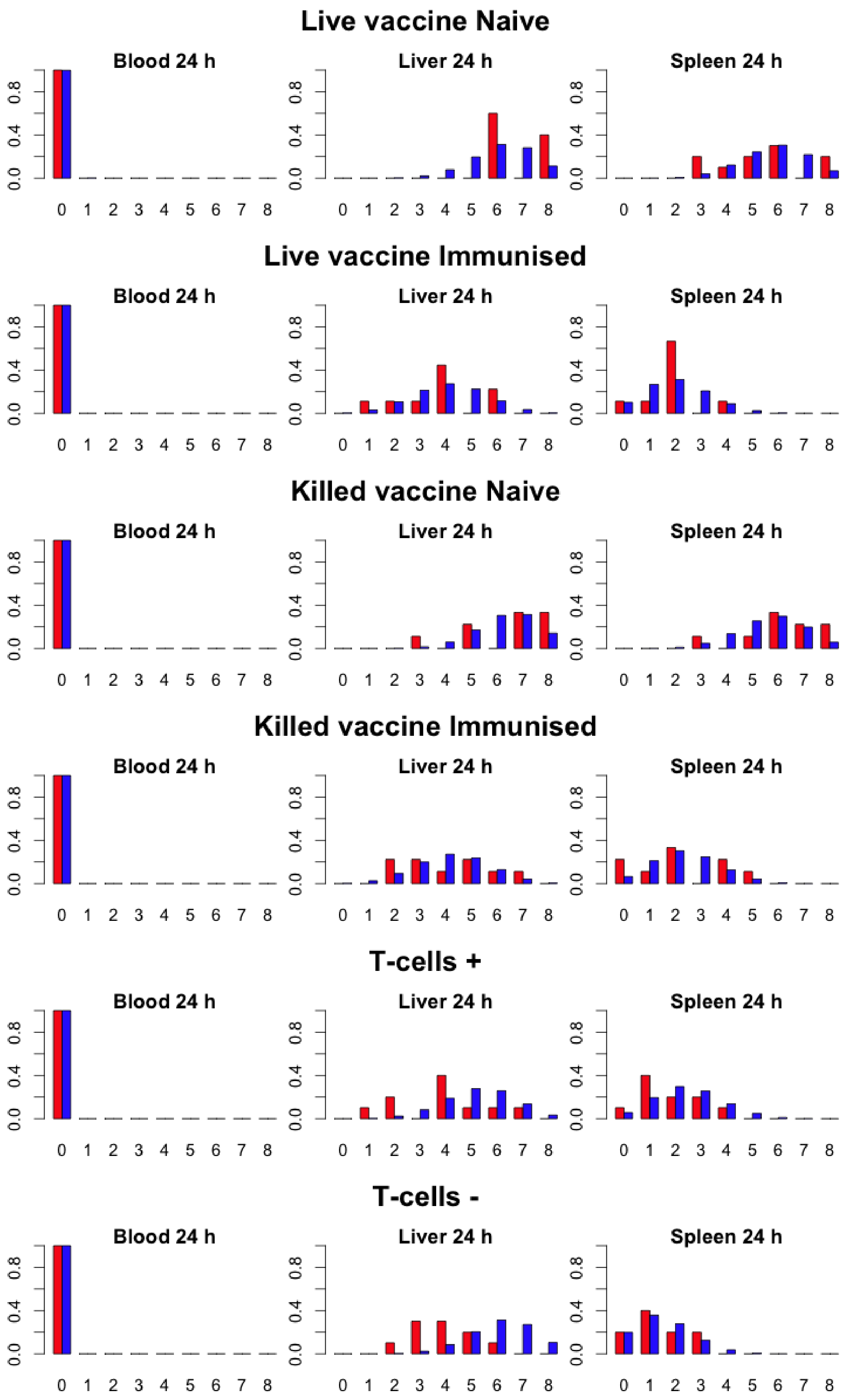

Figure A12. Histograms of the number of WITS present in each compartment.

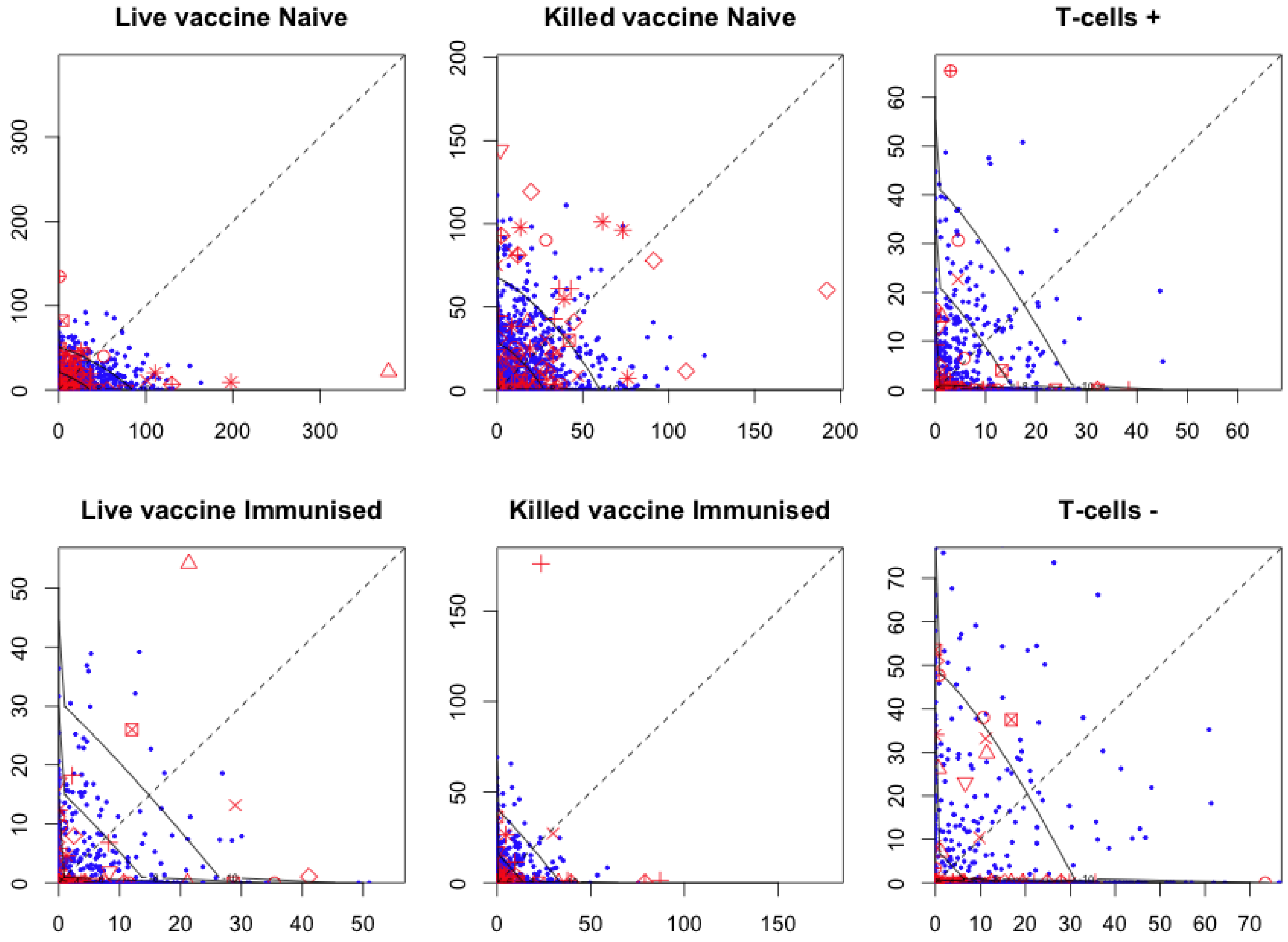

Figure A13. Abundance of WITS in the liver and spleen.

Correlation coefficients between liver and spleen at 24h predicted by the fitted models (see Fig. S1 and S2 for comparison with data):

```
      Live vaccine Naive   Live vaccine Immunised     Killed vaccine Naive
               -0.011481                -0.001850                -0.011679
Killed vaccine Immunised                T-cells +                T-cells -
               -0.002463                -0.006472                -0.007963
```

References

[S1] Press, W. H., Teukolsky, S. A., Vetterling, W. T. and Flannery B. P. (2007) Numerical Recipes 3rd Edition. The Art of Scientific Computing. Cambridge University Press, Cambridge.

[S2] Moulay, E. and Baguelin, M. (2005) Meta-dynamical adaptive systems and their applications to a fractal algorithm and a biological model, Physica D: 207,79-90.

[S3] Dorai-Raj, S. (2006) R package 'powell', available from http://cran.r-project.org/web/packages/powell/index.html

[S4] Powell, M. J. D. (2002). “UOBYQA: unconstrained optimization by quadratic approximation.” Mathematical Programming 92: 555-582.
