# Supplementary material for: The Effects of Vaccination and Immunity on Bacterial Infection Dynamics In Vivo
Source: PLoS Pathog. 2014 Sep 18;10(9):e1004359. doi: 10.1371/journal.ppat.1004359 (PMC4169467; doi:10.1371/journal.ppat.1004359)
Supplement: Table S2 — Presence and absence of WITS in LV-immunised and naive mice at various secondary challenge doses. (PDF) [file ppat.1004359.s010.pdf]

Table S2: Presence (+; green) or absence (-; red) of each of eight WITS (designated 1, 2, 11, 13, 17, 19, 20 and 21) in the spleens and livers at 6 hr post-challenge with the indicated challenge dose. Animals were either previously vaccinated with live-attenuated *Salmonella* SL3261 (LV) or naive controls. *Composition of this sample could not be determined due to overgrowth of the sample with a contaminating organism.

Dose (CFU)	Treatment	Mouse	Spleen 1	Spleen 2	Spleen 11	Spleen 13	Spleen 17	Spleen 19	Spleen 20	Spleen 21	Liver 1	Liver 2	Liver 11	Liver 13	Liver 17	Liver 19	Liver 20	Liver 21
90	Naive	1	-	-	+	-	-	+	+	-	+	+	-	+	+	+	+	+
		2	+	+	+	-	+	+	+	+	-	+	-	+	+	-	-	+
		3	+	+	+	-	-	-	-	-	-	+	-	-	+	+	+	+
		4	+	-	+	-	+	-	+	+	-	+	+	+	+	+	+	-
	LV	1	+	-	-	-	-	-	+	-	-	-	-	-	+	+	-	-
		2	+	-	-	-	-	-	-	-	+	-	+	-	-	-	+	-
		3	-	+	-	-	-	-	+	-	-	+	+	-	-	+	+	-
		4	Not determined*								-	-	-	+	+	+	+	-
300	Naive	1	-	+	+	+	-	+	+	+	+	+	+	+	+	+	-	+
		2	+	+	+	+	+	+	+	+	+	+	+	+	+	+	+	+
		3	+	+	+	+	+	+	+	+	+	+	+	+	+	+	+	+
		4	+	+	+	+	+	+	+	+	+	+	+	+	+	+	+	+
		5	+	+	+	+	+	+	+	+	+	-	+	-	+	+	+	+
		6	+	-	-	+	-	-	+	-	+	+	-	+	+	+	+	+
	LV	1	+	-	+	-	+	+	+	+	+	+	+	+	+	+	+	+
		2	+	-	+	+	+	-	-	-	+	+	+	+	+	+	+	+
		3	-	-	+	+	-	-	-	-	+	-	+	+	+	+	+	+
		4	-	-	-	-	+	-	-	+	+	+	+	+	+	+	+	+
		5	+	-	+	+	-	+	+	-	-	-	+	+	+	-	-	-
		6	+	-	-	+	-	-	+	+	+	+	+	+	+	+	+	+
900	Naive	1	+	+	+	+	+	+	+	+	+	+	+	+	+	+	+	+
		2	+	+	+	+	+	+	+	+	+	+	+	+	+	+	+	+
		3	+	+	-	+	+	+	+	+	+	+	+	+	+	+	+	+
		4	+	+	+	+	+	+	+	+	+	+	+	+	+	+	+	+
	LV	1	+	-	+	+	+	+	+	+	+	+	+	+	+	+	+	+
		2	+	+	+	-	-	-	+	-	+	+	+	+	+	+	+	+
		3	+	+	+	+	+	+	+	+	+	+	+	+	+	+	+	+
		4	+	-	+	-	-	+	-	+	+	+	+	+	+	-	+	+
9000	Naive	1	+	+	+	+	+	+	+	+	+	+	+	+	+	+	+	+
		2	+	+	+	+	+	+	+	+	+	+	+	+	+	+	+	+
		3	+	+	+	+	+	+	+	+	+	+	+	+	+	+	+	+
		4	+	+	+	+	+	+	+	+	+	+	+	+	+	+	+	+
	LV	1	+	+	+	+	+	+	+	+	+	+	+	+	+	+	+	+
		2	+	+	+	+	+	+	+	+	+	+	+	+	+	+	+	+
		3	+	+	+	+	+	+	+	+	+	+	+	+	+	+	+	+
		4	+	+	+	+	+	+	+	+	+	+	+	+	+	+	+	+
